# P2-HNF4α alters linoleic acid metabolism and mitigates soybean oil-induced obesity: role for oxylipins

**DOI:** 10.1016/j.jlr.2025.100932

**Published:** 2025-10-28

**Authors:** Poonamjot Deol, Johannes Fahrmann, Dmitry Grapov, Jun Yang, Jane R. Evans, Oliver Fiehn, Brett Phinney, Bruce D. Hammock, Frances M. Sladek

**Affiliations:** 1 Department of Molecular, Cell and Systems Biology, University of California, Riverside, California, USA; 2 Department of Microbiology and Plant Pathology, University of California, Riverside, California, USA; 3 Department of Clinical Cancer Prevention, The University of Texas MD Anderson Cancer Center, Houston, Texas, USA; 4 CDS - Creative Data Solutions, Colfax, California, USA; 5 Department of Entomology and Nematology & UCD Comprehensive Cancer Center, University of California, Davis, California, USA; 6 Genome Center, University of California, Davis, California, USA

**Keywords:** High-Fat Diet, Metabolic Disease, PUFAs, CYP450, Epoxide Hydrolases, Mitochondrial Function, Metabolomics, Proteomics, Ketone Bodies, TCA Cycle, Proinflammatory cytokines

## Abstract

Oxylipins—oxidized metabolites of polyunsaturated fatty acids (PUFAs)—are associated with several pathological conditions. We previously showed that oxylipin metabolites of linoleic acid (LA) and alpha-linolenic acid positively correlate with obesity in wild-type (WT) mice fed a high fat diet (35% kcal fat) based on soybean oil (SO). Here, we compare the effect of the SO diet (10% kcal LA) to an isocaloric diet based on coconut oil (CO) that is low in LA (2% kcal) in HNF4α exon swap male mice that express only the P2 form of HNF4α (α7HMZ). α7HMZ mice gained significantly less weight on the SO diet than WT mice and exhibited neither glucose intolerance nor fatty liver as did the WT mice. Untargeted metabolomics of the liver revealed increased levels of LA and decreased levels of PUFA-derived C18 diols in α7HMZ compared to WT. Proteomics identified decreased levels of several enzymes involved in PUFA metabolism (CYP2Cs, EPHX1, FADS2, ACOX1/2) as the likely cause of decreased diols. Correlation analysis of hepatic oxylipins with body weight, coupled with a 16-weeks treatment with a soluble epoxide inhibitor (sEHI), identified the oxylipins most likely to be potential drivers of obesity as 9,10-DiHOME, 12,13-DiHOME, 9,10-DiHODE and 12,13-DiHODE. Hepatic accumulation of omega-6 and omega-3 oxylipin metabolites of the essential fatty acids, linoleic and alpha-linolenic, are necessary but not sufficient for diet-induced obesity.

Over the past century there have been major shifts in global dietary patterns, with one of the most notable being the increased consumption of polyunsaturated fats (PUFAs). In the United States, a major source of these PUFAs is soybean oil (SO), which is rich in linoleic acid (LA, C18:2), an essential omega-6 (ω-6) fatty acid that must be obtained from the diet. While relatively small amounts of LA (1–2 kcal%) are required for human health, the remarkable increase in consumption of soybean oil (SO) in the U.S. in the past 50 years has resulted in a notably higher intake of LA (15–25 kcal%) (1, 2). Numerous studies suggest that increased dietary LA (or ω-6 fatty acids in general) and/or its downstream bioactive metabolites (referred to as oxylipins) could be contributing to the development of various metabolic and inflammatory diseases, including the epidemic of obesity in the U.S. (3, 4, 5, 6). Indeed, a previous study from our lab revealed a positive correlation between the increased prevalence of hepatic oxylipins generated from LA and alpha-linolenic acid (ALA) with SO-induced obesity in male mice (7).

LA is the endogenous ligand for the nuclear receptor Hepatocyte Nuclear Factor 4 alpha (HNF4α), a master regulator of liver-specific gene expression (8, 9). HNF4α, a highly conserved transcription factor linked to hemophilia, diabetes, inflammatory bowel disease (IBD) and cancer, is expressed in the liver, intestines, kidney, stomach and pancreas (10, 11). It plays an important role in metabolic functions, including fatty acid oxidation, in HNF4α-expressing as well as non-HNF4α-expressing tissues, such as adipose tissue (12, 13).

The human *HNF4A* gene has two promoters, P1 and P2, that drive the expression of several different isoforms of HNF4α, of which HNF4α1(driven by the P1 promoter) and HNF4α7 (driven by the P2 promoter) are the most predominant (Fig. 1A). This promoter structure and the resulting isoforms are highly conserved across all mammals (10). Expression of the HNF4α isoforms can vary both spatially (14) and temporally (15), and a proper balance of their expression is important for optimal health and function of the tissue (10, 16). Indeed, variants in the HNF4α P2 promoter have been associated with increased risk of metabolic syndrome and late-onset diabetes (17, 18). We and others have shown that high-fat diets can perturb the balance between the P1 and the P2 promoters, leading to increased P2-HNF4α in both the liver (15, 19) and colon (4). Taken together, these findings indicate that HNF4α may play a role in diet-induced obesity and metabolic syndrome, and that the isoforms may play distinct roles (13).

We previously reported that a diet high in soybean oil can cause obesity in wild-type (WT) male mice that express primarily HNF4α1/2 in the liver (20). We also found that the expression of certain cytochrome P450 (*Cyp*) genes that metabolize LA to oxylipins is significantly decreased in the livers of exon-swap mice that express only the P2-isoform of HNF4α (henceforth referred to as α7HMZ) (16, 21). Thus, to determine whether PUFA-derived oxylipins play a causal role in obesity, we subjected the α7HMZ male mice to an SO diet containing 10% LA and performed untargeted metabolomic and proteomic analysis of their livers. The α7HMZ mice were significantly less obese than WT controls and had lower levels of several oxylipins, including the LA-derived 9,10-DiHOME and 12,13-DiHOME as well as the ALA-derived 9,10-DiHODE and 12,13-DiHODE However, they also accumulated more LA than WT mice, consistent with drastically decreased levels of CYP2C enzymes that metabolize LA. The specificity of the effects of the LA-enriched SO diet was underscored by an isocaloric diet based on coconut oil (2% LA) which induced similar weight gain in WT and α7HMZ mice, albeit reduced compared to the SO diet. Finally, while there was no indication of elevated levels of pro-inflammatory cytokines in the livers of the WT or α7HMZ mice, levels of several key metabolites involved in mitochondrial function were elevated in the α7HMZ livers which could also contribute to the lean phenotype.

## Materials and Methods

### Diets

Two isocaloric diets with 40 kcal% fat were formulated in conjunction with Research Diets, Inc. (Supplemental Table S1, in Supplemental Materials file). These diets were: SO+CO (21 kcal% fat calories from coconut oil and 19 kcal% from soybean oil, of which 10 kcal% are from LA); CO (36 kcal% from coconut oil and 4 kcal% from soybean oil to provide the essential fatty acids LA and ALA); standard lab chow (5001 Laboratory Rodent Diet, Newco Distributors) referred to as vivarium chow (Viv) was included as a low fat control.

### Animals

Male C57BL6/N (Charles River Laboratories) or exon swap HNF4α (α7HMZ mice in the C57Bl/6N background) (21) were weaned at three weeks of age and assigned randomly to one of the diets. While the WT and α7HMZ mice are not littermates, the breeding, housing and animal husbandry conditions were identical for both. The α7HMZ line is maintained in a C57BL6/N background using heterozygous matings to generate α7HMZ breeders (from different parents). Only the first generation of those α7HMZ breeders are used to generate mice for the experiments. The whole strain is backcrossed into C57BL6/N mice from Charles River every three to five years to counter any effects from genetic drift. The animals were maintained on a 12:12 h light-dark cycle in a specific pathogen-free (SPF) vivarium. At least 12 mice of each genotype were put on each diet with three to four animals per cage. Food intake was recorded twice a week on a per cage basis; individual mouse weights were recorded once a week for up to 35 weeks when they were euthanized and their tissues collected.

### Ethics statement

Care and treatment of animals was in accord with guidelines from and approval by the University of California, Riverside Institutional Animal Care and Use Committee (AUP#20140014). All mice had ad libitum access to food and water (other than the indicated fasting times). At the end of the study, mice were euthanized by carbon dioxide inhalation, in accordance with NIH guidelines.

### Glucose and insulin tolerance tests

Glucose tolerance (GTT) and insulin tolerance tests (ITT) were performed on WT and α7HMZ mice used in this study had been on the diets for 20 and 33 weeks, respectively (20). Briefly, to measure glucose tolerance (GTT), mice were fasted overnight (∼18 h) and glucose (2 g/kg body weight) was administered by intraperitoneal (IP) injection of a 20% glucose solution in 0.9% saline. Tail blood glucose was measured at 0 (pre-injection), 15, 30, 60 and 120 min after injection using OneTouch Ultra Glucose Meter and OneTouch Ultra Test Strips (LifeScan Inc). To measure insulin sensitivity (ITT), mice were fasted for 4.5 h and then injected IP with 0.75 U/kg of Humulin R (Eli Lilly and Company). Tail blood glucose was measured at 0, 15, 30, 60 and 90 min as for the GTT.

### Tissue samples and staining

Liver was collected, stored and analyzed by Oil Red O staining as described previously (20).

### Multiplex cytokine assay

Cytokine concentrations in liver tissue from mice on the various high-fat diets were measured using the MILLIPLEX® Mouse Cytokine/Chemokine Magnetic Bead Panel (MCYTMAG-70K-PX32, Millipore) according to the manufacturer's instructions. Data were collected using the Luminex-200 system (Life Technologies) housed in the UCR Stem Cell Core and analyzed with the MILLIPLEX® Analyst 5.1 software from EMD Millipore.

### Soluble epoxide hydrolase inhibitor administration

As described previously (22), the sEHI 1-trifluoromethoxyphenyl-3-(1-propionylpiperidin-4-yl) urea (TPPU) (23, 24) was dissolved in polyethylene glycol 400 (PEG-400, Sigma Aldrich) to prepare a clear stock solution at a concentration of 1000 mg/L. This stock solution was subsequently diluted into warm drinking water with vigorous stirring to achieve a final concentration of 10 mg/L TPPU in 1% PEG-400. The TPPU-containing water was supplied to the animals ad libitum.

### Metabolomic, lipidomic, oxylipin, and proteomic analysis

Liver tissue from the large lobe was snap frozen and stored in liquid nitrogen. The MiniX database (25) was used as a Laboratory Information Management System (LIMS) and for sample randomization prior to all analytical procedures. All samples were analyzed in one batch. For analysis of primary metabolites, 5 mg of liver tissue homogenate were extracted and derivatized; metabolite levels were quantified by chromatography time-of-flight (GC-TOF) mass spectrometry as previously described (26). The precipitated protein from the primary metabolite analysis was used for the proteomic analysis. For analysis of complex lipids, liver tissue homogenates (5 mg) were extracted using a modified liquid-liquid phase extraction approach (27). For analysis of non-esterified oxylipins, liver tissue homogenates (100 mg) were extracted and analyzed according to previously described protocols (27, 28, 29).

Details on the sample collection and metabolomic and proteomic analysis have been described previously (7). A total of 369 primary metabolites were detected of which 151 were known and 218 were unknown metabolites. For complex lipids, the total number detected was 3238, with 273 known and 2965 unknown. See Supplemental Table S2 for all the datasets, including separate datasheets for primary metabolites, complex lipids, oxylipins and proteomics results for the liver, as well as information sheets for each platform. The raw metabolomics data have been deposited on the Metabolomics Workbench (www.metabolomicsworkbench.org) under Project # PR000461. Proteomics data for liver tissue have been deposited in the proteomics repository Massive (http://massive.ucsd.edu) with ID# MSV000081149; it can also be accessed via Proteome Exchange with ID # PXD006681.

### Statistical analysis

Data are presented as mean ± standard error of mean (SEM). Statistical significance, using PRISM (version 10.0.1 for Mac; www.graphpad.com), is defined as *P* ≤ 0.05 using the following tests: Two-way ANOVA with Holm-Sidak post hoc analysis for differences in weight gain over time among the different diets. Student's *t* test was performed for tissue weights at harvest. One-way ANOVA was used for the GTT and ITT assays. For metabolomics data, Student's *t* test was used to determine significantly different metabolites between two groups. When comparing more than two groups in an analysis, values were log2 transformed and statistical significance was determined using a one-way ANOVA. Specific group differences were determined using Tukey HSD post hoc test. ANOVA *P*-values were adjusted using Benjamini and Hochberg false-discovery rate adjustment. The PRISM pairwise comparison function was used to depict lines (and asterisks) above the bars to indicate pairs of groups that showed statistically significant differences in the post-hoc analysis. The lines above the bar graphs start and end with the comparisons that are significantly different. Statistical analyses for metabolomics data were conducted using R statistical software. For correlations between metabolites and metabolic phenotypes, Spearman's rank correlations on log10-transformed values of known compounds were performed; only significant correlations are included (*P* < 0.05) (Supplemental Fig. 6). Linear regression analysis was performed between body weight and concentration of metabolites in the liver. The following cut-offs were used to determine significance: Spearman's coefficient r > 0.5 with *P* < 0.05 and *R*^2^ > 0.5. For major structural lipids, the summed intensities of all lipids belonging to that specific lipid class (e.g., triacylglycerides) were used. Lipids were delineated by degree of saturation. Saturated: <2 or <4 double bonds present in lipid species that contain one or more acyl chains, respectively. Unsaturated: ≥2 or ≥4 double bonds present in lipid species that contain one or more acyl chains, respectively. Unless otherwise noted in the figure legend, all graphs presented in the manuscript were plotted using PRISM. For these graphs, asterisks indicate significance based on one-way ANOVA across both genotypes and all three diets; *P* values, wherever included on graphs, are for difference between the two indicated groups based on Student's *t* test. Unless indicated otherwise, heatmaps were generated with the Heatmaps function in PRISM.

### Pathway impact analysis

A significant difference in expression between WT and α7HMZ livers was observed for 45 primary metabolites. Pathway enrichment analysis was performed on these metabolites using the pathway analysis module in Metaboanalyst 5.0 (30). This module performs the enrichment analysis based on pathways identified in the Kyoto Encyclopedia of Genes and Genomes (KEGG) database (31). See Supplemental Materials for details on the analysis.

## Results

### α7HMZ mice are resistant to soybean oil (SO)-induced obesity, glucose intolerance and fatty liver

**Fig. 1:**
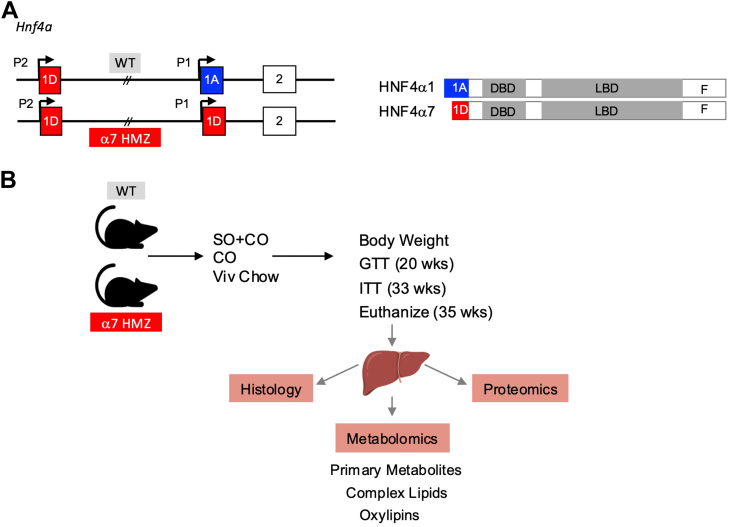
Structure of *Hnf4a* promoters and workflow of experiments performed. A: *Hnf4a* P1 and P2 promoters and first exons in WT and α7HMZ mice, protein products (P1: HNF4α1; P2: HNF4α7). DBD, DNA-binding domain; LBD, ligand-binding domain. Normal adult WT mice primarily express P1-HNF4α in the liver. B: Work-flow: male C57Bl/6N or α7HMZ male mice were weaned at 3 weeks of age to either a low-fat diet (Viv) or one of the two high-fat diets—coconut oil (CO) or soybean oil (SO + CO) enriched. Body weight was measured every week until euthanasia, while other procedures were conducted at the indicated time points. Liver samples were collected for histology, metabolomics and proteomics. See Supplemental Table S1 (in the Supplemental Materials file) for diet composition.

WT male mice that express predominantly P1-HNF4α in the liver and α7HMZ mice that express only the P2-HNF4α isoform (Fig. 1A) were subjected to a high fat diet based on soybean oil that is high in LA (SO+CO, 40% kcal fat, 10% kcal LA) as well as an isocaloric diet made with coconut oil that is low in LA (CO, 2% kcal LA) and a low fat diet (Viv, 13% kcal fat, 1.2% LA). Body weight, glucose and insulin tolerance were tracked, and liver histology, metabolome and proteome were analyzed after 20–35 weeks on the diets (Fig. 1B).

**Fig. 2:**
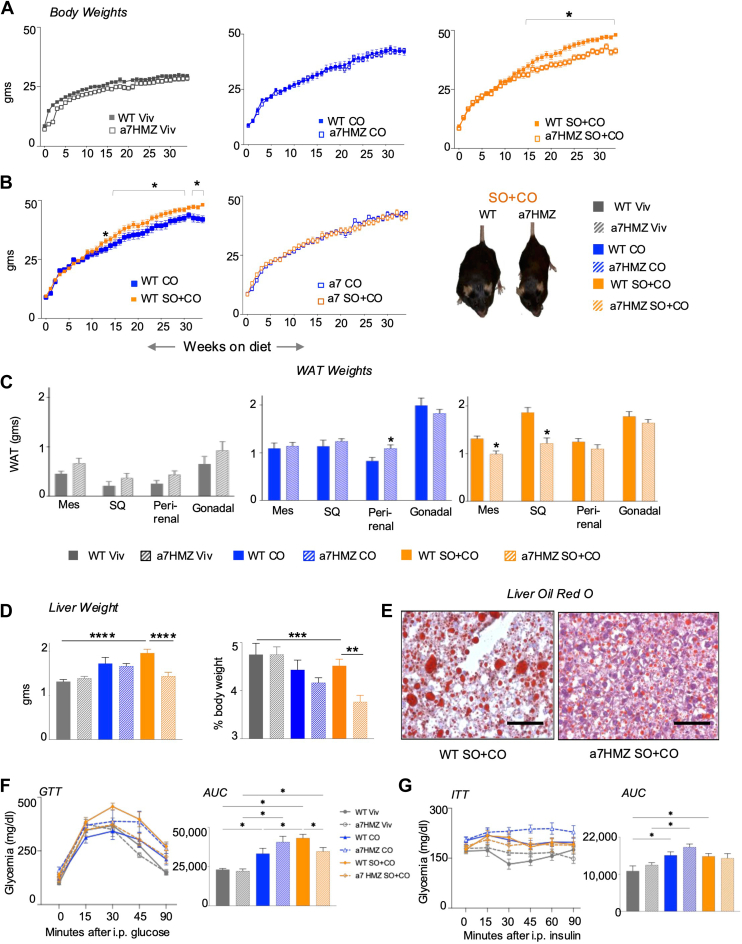
α7HMZ mice are resistant to soybean oil (SO)-induced obesity, fatty liver and glucose intolerance. A: Comparison of average weekly body weights of male C57BL/6N and α7HMZ mice on low-fat diet (Viv) and 40 kcal% high fat diets: CO, coconut oil; SO + CO, soybean oil-enriched; N = 9–13 mice per group. B: Comparison of average weekly body weight between two diets as in (A) but within the same genotype; N = 12–13 mice per group. Photographs of representative mice after 24 weeks on the diet. C: Average weight of white adipose tissues. (WAT; Mes - mesenteric; SQ - subcutaneous). N = 9–13 mice per group. Define Mes and SQ D: Average liver weight in grams and as percent of body weight. N = 9–13 mice per group. E: Representative Oil Red O staining of livers from mice on the SO+CO diet for 35 weeks. Scale bar is 100 μm (See Supplemental Figure S2 for additional stains for both WT and α7HMZ livers and references 7 and 20 for additional ORO stains from similarly treated WT mice). F and G: Glucose excursion curves and area under the curve (AUC) for glucose tolerance test (GTT) at 20 weeks (F) and insulin tolerance test (ITT) at 33 weeks (G) on the diets. N = 3–12 mice per group. A–D and F, G. Data are presented as ± SEM. Significantly different ∗(*P* < 0.05), ∗∗(*P* < 0.01), ∗∗∗(*P* < 0.001) by one-way ANOVA (Benjamini Hochberg post-hoc analysis). The lines above the bar graphs start and end at the comparisons that are significantly different.

The results show that the α7HMZ mice on the low fat and CO diet gained the same amount of body weight as the WT mice, indicating that the α7HMZ mice do not have an inherent growth defect and are capable of absorbing and storing excess dietary fat. In contrast, the α7HMZ SO+CO mice gained significantly less body weight than WT mice on SO+CO (Fig. 2A). The amount of weight gained by the α7HMZ mice on the CO and SO+CO diets was essentially identical (395% and 386% weight gain respectively, from week 0), unlike the WT mice which gained significantly more weight on the SO+CO diet (411%) than the CO diet (370%) (Fig. 2B, Supplemental Fig. 1A, B). The amount of white adipose tissue (WAT) accumulation, particularly in mesenteric and subcutaneous depots was also significantly lower in the α7HMZ mice on SO+CO compared to WT (Fig. 2C, Supplemental Fig. 1C, D). There was no difference in WAT weights between the two genotypes on the low-fat diet although the α7HMZ mice on the CO diet accumulated slightly more perirenal fat than WT (Fig. 2C). Interestingly, the kidney weight was lower in α7HMZ mice regardless of diet, consistent with the P1-HNF4α being the primary isoform in the kidney (10) (Supplemental Fig. 1E). Importantly, there was no difference in the number of kcals consumed by mice on the different diets (Supplemental Fig. 1F).

The α7HMZ mice fed the SO+CO diet had significantly decreased liver: body weight ratio compared to the WT mice as well as compared to chow-fed α7HMZ; histological analysis showed smaller fat droplets in the α7HMZ livers (Fig. 2D, E). The CO-fed α7HMZ and WT mice also had very little liver fat accumulation, similar to what we have observed previously (7, 20) (Supplemental Fig. 2A).

Interestingly, while only SO+CO increased glucose intolerance in WT mice compared to the Viv diet, both high-fat diets did so in α7HMZ mice (Supplemental Fig. 2B) although the α7HMZ mice on SO+CO diet were less glucose intolerant than WT mice (Fig. 2F). Finally, insulin resistance was increased in α7HMZ mice on the CO diet, but not the SO+CO diet. In WT mice, both CO and SO+CO increased insulin resistance compared to chow-fed mice (Fig. 2G, Supplemental Fig. 2C), previously, only the SO+CO diet increased insulin resistance in WT mice (20).

### Metabolomic analysis reveals differential impact of HNF4α isoforms on lipid metabolism and mitochondrial function

**Fig. 3:**
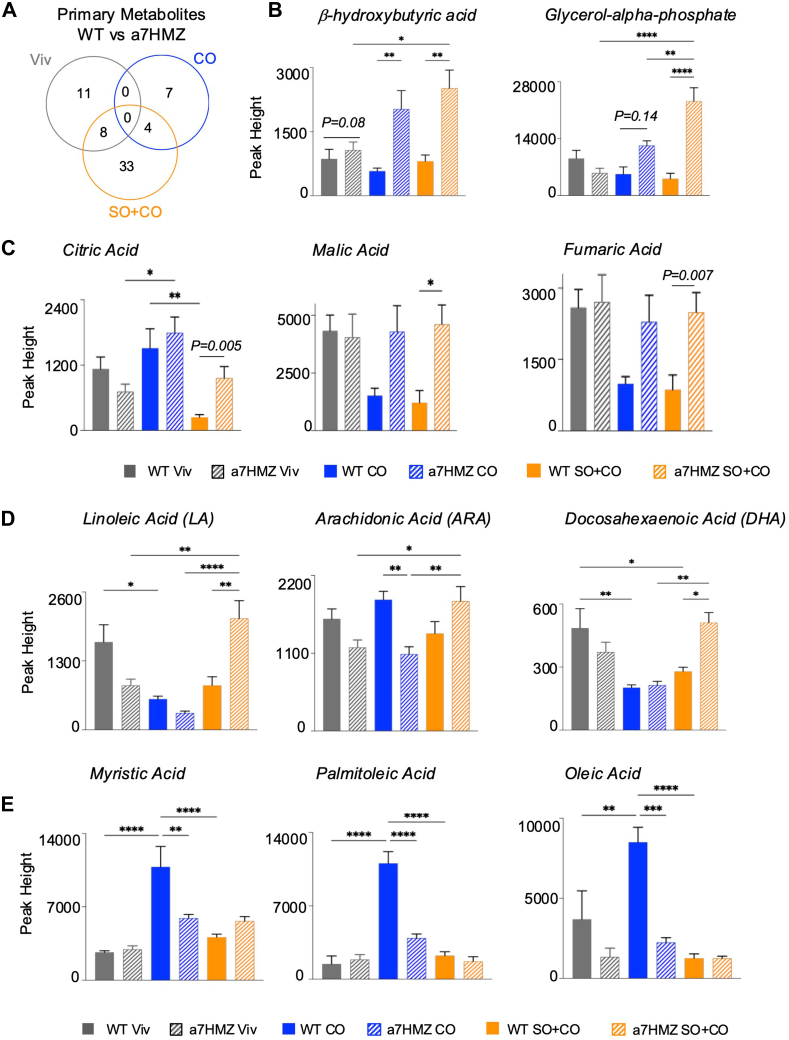
**Metabolomic analysis reveals differential impact of HNF4α isoforms on lipid metabolism and mitochondrial function in the liver.** A: Venn diagram showing the overlap of primary metabolites (liver) that are significantly different between WT and α7HMZ mice in the indicated diet. B–E: Levels of indicated metabolites in the livers of mice fed the respective diets for 35 weeks. Data are presented as ± SEM. Significantly different ∗(*P* < 0.05), ∗∗(*P* < 0.01), ∗∗∗(*P* < 0.001) by one-way ANOVA (Benjamini Hochberg post-hoc analysis). *P* value, when given in the graph, is based on Student's *t* test between the indicated comparisons. N = 7–8 mice per group.

Metabolomic analysis of the liver revealed a total of 45 primary metabolites that were significantly different between the livers of WT and α7HMZ SO+CO-fed mice, of which 33 were unique to the SO+CO diet. In contrast, there were only 11 metabolites uniquely different in chow-fed mice and seven in the CO-fed mice (Fig. 3A) Four metabolites—3-hydroxybutyric acid (a ketone body), glycerol-alpha-phosphate (also known as glycerol-3-phosphate, an intermediate in the glycolysis metabolic pathway) (https://hmdb.ca/metabolites/HMDB0000126#references), lyxitol (also known as L-arabitolis is a sugar alcohol with glycosidase inhibitory activity) and N-methylalanine (an alanine derivative)—were all upregulated in α7HMZ compared to WT mice on both the CO and SO+CO diets, but not in those on the Viv diet, suggesting a combined effect of the HNF4α isoforms and fat intake (Fig. 3B, Supplemental Fig. 3A). Included in the 33 metabolites unique to the SO+CO diet were three intermediates of the tricarboxylic acid (TCA) cycle (citric acid, malic acid and fumaric acid) which were all elevated in α7HMZ livers compared to WT in the SO+CO diet (Fig. 3C). They were all also decreased in WT SO+CO compared to WT Viv or CO diets. In contrast, only citric acid was altered by diet in α7HMZ livers, with an increase observed in the CO versus Viv diet (Fig. 3C, Supplemental Fig. 3B). These metabolites play an important role in energy expenditure, as do 3-hydroxybutyric acid and glycerol-alpha-phosphate, and implicate HNF4α in mitochondrial function.

### Linoleic acid and other fatty acids are differentially regulated by the HNF4α isoforms

Analysis of all 45 SO+CO primary metabolites using MetaboAnalyst identified LA metabolism as one of the pathways with the highest impact (Supplemental Fig. 4A). There was a notable increase in the amount of LA in the liver of α7HMZ mice fed the SO+CO diet compared to chow (Fig. 3D). This accumulation was not observed in the WT mice; in fact, there was a decrease in LA in the SO+CO diet-fed WT mice compared to Viv (Supplemental Fig. 4B). The LA metabolite arachidonic acid (ARA, C20:4) had a similar profile to LA in that ARA was increased in α7HMZ SO+CO compared to CO and chow (Fig. 3D). The omega-3 fatty acid, linolenic (ALA, C18:3), did not show a significant difference in hepatic accumulation between any of the groups (Supplemental Fig. 4C). Eicosapentaenoic acid (EPA, C22:5n-3) was highest in the low-fat diet in both genotypes. In contrast, docosahexaenoic acid (DHA, C22:6n-3) showed a similar pattern of accumulation as LA with WT chow and α7HMZ SO+CO livers having the highest levels and the CO diet the lowest levels (Fig. 3D). This is consistent with the well-established role of DHA as an anti-inflammatory compound (32).

The fatty acids that had the greatest accumulation in WT mice on the CO diet were myristic (C14:0), palmitoleic (C16:1) and oleic acid (C18:1) (Fig. 3E). Myristic acid is one of the most abundant fatty acids in coconut oil, while palmitoleic acid is derived from palmitic acid (C16:0) which is also abundant in coconut oil, and can be converted to oleic acid, the major fatty acid in olive oil, a key component of the healthy Mediterranean diet. Notably, this conversion to oleic acid seemed to be repressed by the presence of soybean oil in the SO+CO diet (Fig. 3E). Interestingly, none of these fatty acids showed increased accumulation in the α7HMZ mice while ketone bodies (β-hydroxybutyric acid, also known as 3-hydroxybutyric acid), which are derived from free fatty acids including myristic, palmitoleic and oleic acid, were increased in the α7HMZ mice fed the CO diet (Fig. 3B, E).

### Hepatic levels of complex lipids vary with both diet and genotype

**Fig. 4:**
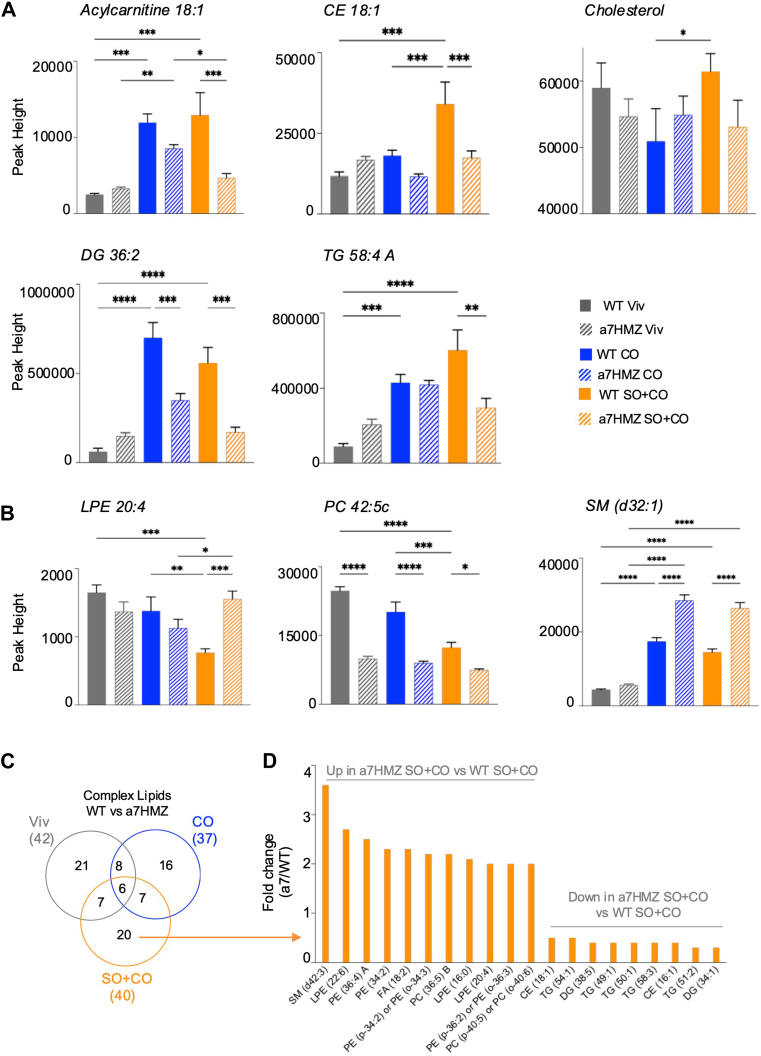
Metabolomic analysis reveals differential impact of HNF4α isoforms on fatty acid accumulation in liver. A, B: Levels of the indicated metabolites in the livers of mice fed the respective diets for 35 weeks. Data are presented as ± SEM. ∗Significantly different (*P* < 0.05) by one-way ANOVA. C: Venn diagram showing the overlap of complex lipid species that are significantly different between WT and α7HMZ mice in the indicated diet comparisons. D: Bar graph showing fold-change in levels of 20 complex lipids significantly different (*P* < 0.05, fold change ≥ 2) between WT and α7HMZ on the SO+CO diet. Significantly different ∗(*P* < 0.05), ∗∗(*P* < 0.01), ∗∗∗(*P* < 0.001) by one-way ANOVA (Benjamini Hochberg post-hoc analysis). (See Supplemental Table 2 for additional comparisons.)

Having observed differences in free fatty acids, we next examined the abundance of complex lipids in the livers of the WT and α7HMZ mice fed the three diets. Supplemental Figure 5A shows the cumulative levels of the various annotated complex lipid species while Fig. 4A, B shows representative individual compounds: acylcarnitines (AC), ceramides, cholesteryl esters (CE), diacylglycerols (DG), glyceroceramides (GlcCer), lysophosphatidylcholines (LPC), lysophosphatidylethanolamines (LPE), phosphatidylcholines (PC), phosphatidylethanolamines (PE), phosphatidylinositols (PI), sphingomyelins (SM), and triacylglycerols (TG). In WT mice both CO and SO+CO diets increase DG, TG, CE although the soybean oil diet increases them more than the coconut oil diet. In contrast, in α7HMZ mice the most notable increase above the low-fat diet is the TGs. Levels of acylcarnitines in α7HMZ mice are also increased on the low-fat diet compared to WT mice, which could contribute to improved mitochondrial function. Interestingly, LPEs, which have been associated with improved mitochondrial function in the heart (33), are decreased by the SO+CO diet in WT mice but not in α7HMZ mice. Hepatic cholesterol levels follow the same trend with cholesteryl esters in WT mice on SO+CO showing the highest level, notably higher than the CO diet which consist largely of saturated fats (Fig. 4A, Supplemental Fig. 5A). Some other individual lipids that show large differences between WT and α7HMZ mice are AC 18:1, DG 36:2, LPE 20:4, PC 42:5 and SM(d32:1) (Fig. 4 B, C). It is worth noting that not all of the individual compounds belonging to a lipid family follow the overall trend—e.g., TG 58:3 has the highest levels in WT mice on the CO diet while TG 50:2 is increased by the CO diet in both WT and α7HMZ and the SO+CO diet in WT Supplemental Fig. 5B. Overall, the α7HMZ mice on the SO+CO diet have lower total levels of various lipid families, including cholesteryl esters, diacylglycerols and triacylglycerols, which could explain the reduced hepatic steatosis in the α7HMZ livers compared to WT.

There are 40 complex lipids that are significantly dysregulated (*P* < 0 0.05 and >2-fold difference) between α7HMZ and WT mice on the SO+CO diet (Fig. 4C). Of these, six are also dysregulated in the chow versus CO comparison while seven are commonly dysregulated by either Viv or CO diet versus SO+CO. Among the 20 complex lipids that are exclusively altered by SO+CO in α7HMZ versus WT are three LPEs (LPE 16:0, 20:4; 22:6) and linoleic acid (C18:2) that are increased in α7HMZ livers while several CEs (CE 16:1, 18:1), DGs (DG 34:1, 38:5) and TGs (TG 49:1, 50:1, 51:2, 54:1, 58:3) are decreased (Fig. 4D). (See Supplemental Table 2 for all data).

### Livers of soybean oil-fed α7HMZ mice have decreased levels of oxylipins

Spearman Rank Order Correlation plots of all the metabolomic data (primary metabolites, complex lipids, and oxylipins) and phenotypic metrics of obesity (body, kidney, liver and adipose weight, and GTT) across both genotypes (WT, α7HMZ) and all three diets (Viv, CO, SO+CO) revealed that the most numerous positive correlations were between the oxylipin metabolites derived originally from LA and ALA and body weight in WT versus α7HMZ on the soybean oil diet (Supplemental Figs. 6 and 7; see Supplemental Table 2 for additional correlation plots)

**Fig. 5:**
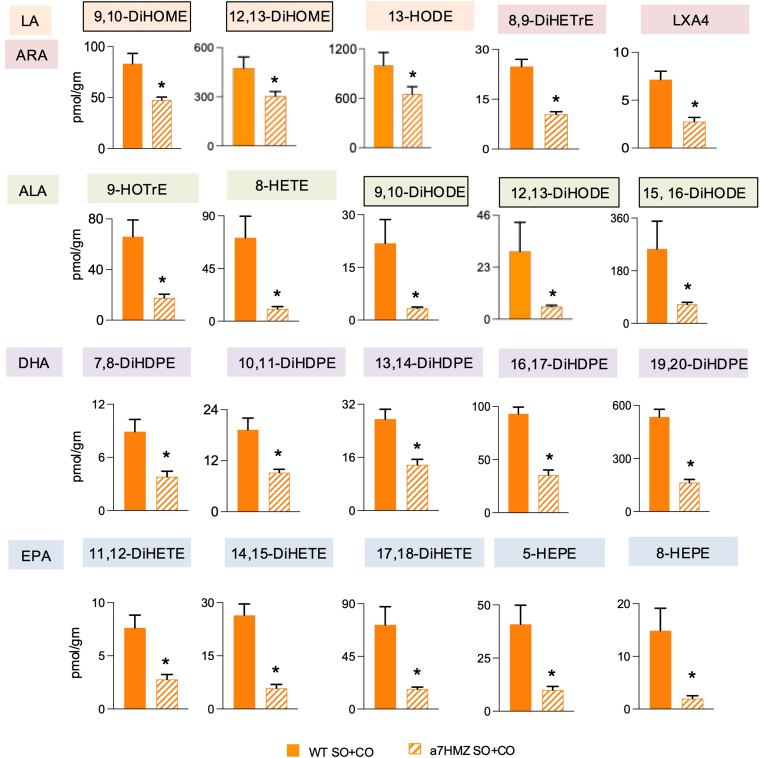
Livers of soybean oil-fed α7HMZ mice have decreased levels of oxylipins. Absolute levels of oxylipins in livers of WT and α7HMZ mice fed SO+CO diet. Parent fatty acid and its oxylipins are color-coded. ∗Significantly different (*P* < 0.05) by Student's *t* test. Data are presented as ± SEM. N = 5 mice per group.

**Fig. 6:**
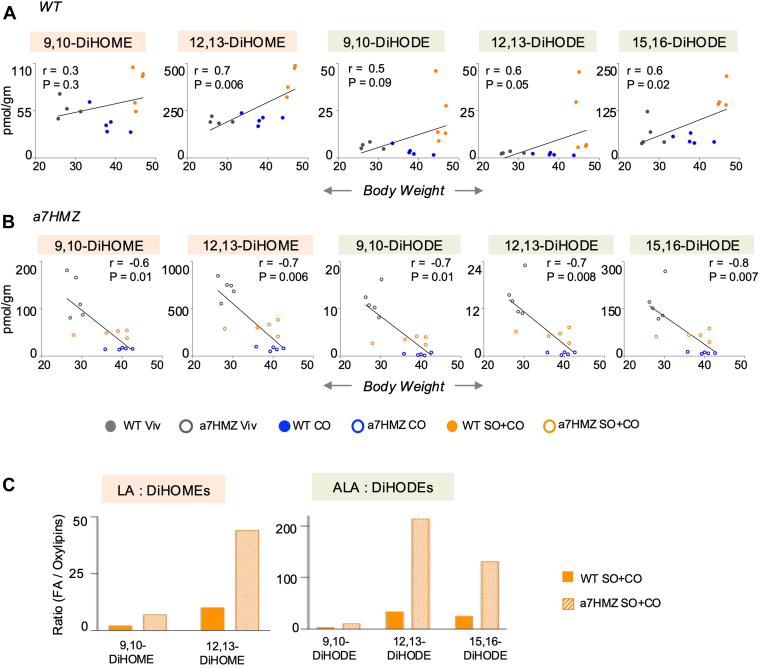
Liver oxylipins correlate with soybean oil-induced obesity. A, B: Correlation between body weight and concentration of liver oxylipins in individual mice fed one of three diets (Viv, CO or SO+CO). Spearman correlation coefficient (r) and *P*-value for each correlation is indicated. C: Ratio of fatty acid to oxylipins derived from it is shown for WT and α7HMZ mice fed SO+CO diet. A–C: N = 5 mice per group. Oxylipins are color-coded by parent fatty acid as in Figure 5.

We previously reported five hepatic oxylipins that correlated positively with obesity in WT male mice after 24 weeks on the SO+CO diet (9,10-DiHODE, 12,13-DiHODE and 15,16-DiHODE from ALA and 9,10-DiHOME and 12,13-DiHOME from LA) (7). Comparison of the levels of these five oxylipins in the livers of α7HMZ and WT mice fed the same SO+CO diet for 35 weeks showed that all five were decreased in α7HMZ livers, along with an additional 15 oxylipins (Fig. 5). Importantly, 12,13-DiHOME and two DiHODEs (12,13- and 15,16-DiHODE) had significant positive correlations (*P* < 0.05, r value = 0.5 to 0.7) with body weight across all three diets in the WT livers; correlations for 9,10-DiHOME and 9,10-DiHODE were not significant but trended in the positive direction (Fig. 6A). In sharp contrast, all five DiHODEs and DiHOMEs correlated negatively with body weight in α7HMZ livers (*P* < 0.01 and r value = −0.6 to to 0.8) (Fig. 6B). Furthermore, the ratio of LA:DiHOME and ALA:DiHODE were much higher in α7HMZ than WT mice on SO+CO diet suggesting that the ratio of the parent fatty acid to its oxylipin metabolites may also be important for countering the obesogenic effects of the soybean oil diet (Fig. 6C). Finally, there were three EPA-derived oxylipins – 8,9-DiHETE, 11,12-DiHETE and 14,15-DiHETE – that showed a negative correlation with weight gain for both genotypes, albeit more so in α7HMZ than WT mice (Supplemental Fig. 8). Consistently, some of these compounds are known to be associated with positive health benefits (34).

### Livers of soybean oil-fed α7HMZ mice have decreased levels of FADS1, ACOX, and CYP450 enzymes

**Fig. 7:**
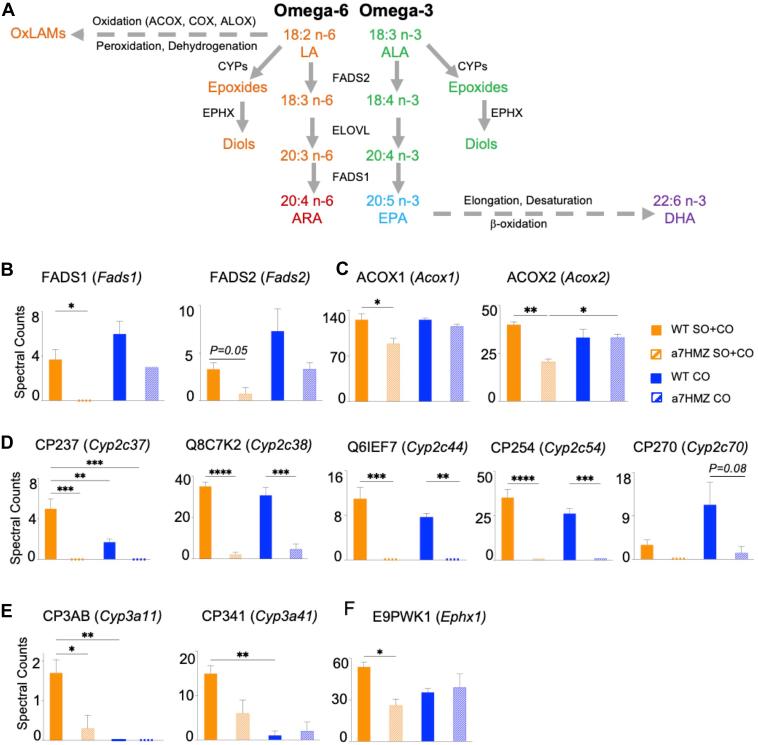
Livers of soybean oil-fed α7HMZ mice have decreased levels of FADS1, ACOX and CYP450 enzymes. A: Schematic showing enzymes and metabolites involved in PUFA metabolism. B–F: Levels of indicated proteins in livers of WT and α7HMZ mice fed either the SO+CO or CO diets. N = 3 mice per group. Data are presented as ± SEM. Significantly different ∗(*P* < 0.05), ∗∗(*P* < 0.01), ∗∗∗(*P* < 0.001) (Tukey’s post-hoc analysis).

The metabolism of long-chain PUFAs to oxylipins involves a series of steps including desaturation by fatty acid desaturase 1 and 2 (FADS1 and FADS2), elongation by fatty acid elongase (ELOVL) enzymes, and oxidation by ACOX, COX and ALOX enzymes to oxidized LA metabolites (OxLAMs) and by cytochrome P450 enzymes (CYP450) to the PUFA epoxides. The final step is hydrolysis to the corresponding diols by epoxide hydrolases (EPHX1, EPHX2) (Fig. 7A). To determine whether there are any alterations in the level of these, or other, proteins in the α7HMZ mice that could explain the SO-resistant phenotype, we performed a proteomic analysis of livers of WT and α7HMZ mice fed either the CO or SO+CO diet (Supplemental Fig. 9). While none of the ELOVL enzymes were altered by either genotype or diet, a significant decrease was observed in the level of FADS1 in α7HMZ mice on SO+CO compared to WT; FADS2 also trended in the same direction (Fig. 7B). ACOX1/2 which are involved in the oxidation of LA into oxidized metabolites called HODEs, were also decreased in α7HMZ livers (Fig. 7C). There was also a remarkable decrease in the levels of several CYPs in α7HMZ versus WT mice on both CO and SO+CO, including five that metabolize both LA and ALA into oxylipins—CP237 (*Cyp2c37*), Q8C7K2 (Cyp2c38), CP254 (Cyp2c54), Q6IEF7 (*Cyp2c44*) and CP270 (*Cyp2c70*) (Fig. 7D, Supplemental Fig. 10). CYPs that metabolize other compounds—including drugs CP3AB (*Cyp3a11*) and CP341 (*Cyp3a41*) (Fig. 7E), retinol (*CYP2a12*) and steroids (CYP7B1, CYP8B1) – were also greatly reduced in α7HMZ livers compared to WT on either the CO or SO+CO diets (Supplemental Fig. 10).

### Livers of soybean oil-fed α7HMZ mice have decreased levels of EPHX1 enzyme resulting in increased levels of epoxides

**Fig. 8:**
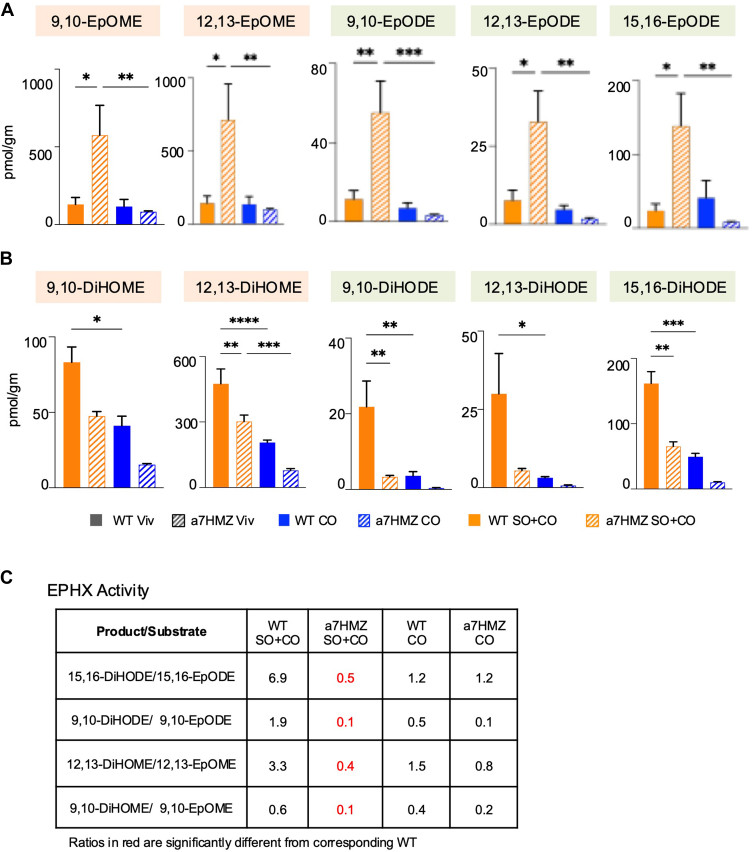
Livers of soybean oil-fed α7HMZ mice have increased levels of epoxides and decreased EPHX activity. A-B: Absolute levels of EPOMEs (A) and their corresponding diols (B) in livers of WT and α7HMZ mice fed either the SO+CO or CO diets. Data are presented as ± SEM. Significantly different ∗(*P* < 0.05), ∗∗(*P* < 0.01), ∗∗∗(*P* < 0.001) by one-way ANOVA. C: Ratio of oxylipin diol:epoxide as a measure of EPHX activity. Red, significantly different from corresponding WT (*P* ≤ 0.05, *t* test). N = 5 per group.

Oxidation of fatty acids by CYP proteins or non-enzymatic action of free radicals generates epoxides which are subsequently hydrolyzed into oxylipins by EPHX1/2 (Fig. 7A). The proteomic analysis revealed decreased levels of EPHX1 in α7HMZ livers compared to WT on the SO+CO diet (Fig. 7F), consistent with increased levels of epoxides in α7HMZ (9,10-EpOME; 12,13-EpOME: 9,10-EpODE; 12,13-EpODE; 15,16-EpODE) and decreased levels of oxylipins (9,10-DiHOME; 12,13-DiHOME: 9,10-DiHODE; 12,13-DiHODE; 15,16-DiHODE) (Fig. 8A, B). Consequently, the overall EPHX activity, defined as the product/substrate ratio of the oxylipin:epoxide, was significantly lower in α7HMZ versus WT on SO+CO (Fig. 8C). It is notable that there was no difference in levels of EPHX1 between WT and α7HMZ on the CO diet consistent with a lack of difference in the product/substrate ratio (Figs. 7F and 8C).

**Fig. 9:**
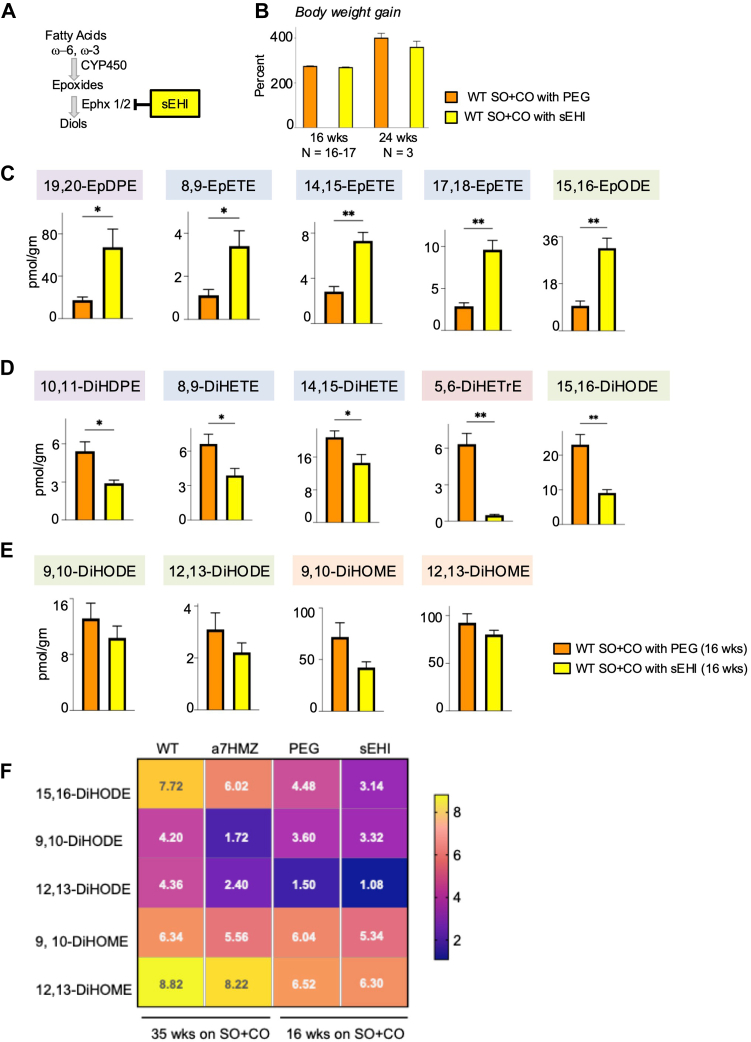
Accumulation of certain oxylipin diols is required for SO-induced weight gain. A: Schematic showing mechanism of action of soluble epoxide hydrolase (sEHI). B: Body weight gain in WT mice fed the SO+CO diet treated with vehicle (PEG) or sEHI in the drinking water for 16 (N = 16–17) or 24 (N = 3) weeks. C, D: Absolute levels of oxylipin epoxides (C) and diols (D) in liver that showed a significant difference between PEG and sEHI treatments of WT mice fed the SO+CO diet for 16-weeks. E. Diols that did not show a significant change in levels in the liver upon sEHI treatment. Data are presented as ± SEM. Significantly different ∗(*P* < 0.05), ∗∗(*P* < 0.01) by one-way ANOVA. F: Heatmap showing Log2 fold expression of hepatic oxylipin diols from Figure 6 that correlated positively with body weight in WT and negatively in α7HMZ mice fed the SO+CO diet for 35 weeks versus their levels in PEG- or sEHI-treated mice for 16 weeks. N = 5 per group.

As another approach to elucidate a potential causative role for specific oxylipins in SO-induced obesity, we treated WT mice on the soybean oil diet with an inhibitor of EPHX2 (sEHI) in the drinking water for 16 weeks and analyzed the oxylipin levels in the liver. Both the sEHI and vehicle control (PEG) gained similar amounts of body weight (Fig. 9A, B). Nonetheless, there well as a notable increase in several epoxides (19,20-EpDPE, 8,9-EpETE, 14,15-EpETE, 17,18-EpETE and 15,16-EpODE) indicating that the sEHI treatment was effective (Fig. 9C). In contrast, there was a significant decrease in several diols by the sEHI treatment (10,11-DiHDPE, 8,9-DiHETE, 14,15-DiHETE, 5,6-DiHETrE and 15,16-DiHODE), suggesting that they do not play a causative role in obesity (Fig. 9D). Importantly, there was no significant change in a second set of diols (9,10-DiHODE, 12,13-DiHODE, 9,10-DiHOME and 12,13-DiHOME) (Fig. 9E, F) that had been identified as correlating positively with obesity (Fig. 6) (7), supporting the notion that they may in fact be helping to drive obesity since the sEHI treatment did not reduce body weight. The only exception is 15,16-DiHODE which decreased in the sEHI livers (Fig. 9D, F) suggesting that it may not be a main driver of SO-induced obesity.

Given that the increase in body weight by the SO diet typically begins at 10 weeks (Fig. 2A, B; Supplemental Fig. 1B), the sEHI results serve as a snapshot of the liver at the beginning of the onset of weight gain which could be more relevant for promoting obesity than a later time point when metabolite levels could be a consequence of the obese state instead of a cause. Notably, a pilot group of mice treated by sEHI for 24 weeks also showed no significant difference in body weight compared to the vehicle control (Fig. 9B). Furthermore, the levels of the vast majority of the oxylipins trended higher in the WT mice fed the SO diet for 35 weeks than the control animals (PEG) in the 16-weeks sEHI experiment, consistent with the notion that the oxylipins accumulate in tissue over time (Fig. 9F, Supplemental Fig. 11). The only exception among the diols correlating positively with obesity is 9,10-DiHOME which showed minimal accumulation between 16 and 35 weeks.

Finally, since obesity is often associated with a pro-inflammatory environment, we analyzed the levels of 32 cytokines in the liver of the WT and α7HMZ mice on the three diets (Viv, CO, SO+CO) (Supplemental Table 3). The only cytokines that showed a significant difference between WT and α7HMZ mice fed the SO+CO diet were IL-3, IL-5, IL-9—all three were lower in α7HMZ livers (Supplemental Fig. 12). However, the levels of these cytokines in the SO+CO diet in WT livers were not significantly greater than in the WT mice fed the low-fat diet. Furthermore, while all three of these cytokines have been found to be elevated in various obese populations in humans (35, 36, 37), other pro-inflammatory cytokines such as IL-1, IL-6 and TNFα were not significantly altered by either diet or genotype, suggesting that at least in the WT mouse liver neither coconut oil nor soybean oil create a pro-inflammatory environment.

## Discussion

Linoleic acid (LA, C18:2ω6) is an essential fatty acid that must be obtained from the diet (38). However, there is a debate about what is the optimal amount of dietary LA, ranging from the minimum required (1–2%kcal) as in a Paleo diet to 5%–10% kcal in the current U.S. dietary guidelines (39, 40). This debate has become increasingly relevant as the use of soybean oil, which is naturally high in LA, for cooking and in processed foods has become a worldwide trend (1, 2, 41). We and others have shown previously that diets high in LA can lead to negative health outcomes, including obesity, diabetes and sensitivity to IBD, and that oxylipin metabolites of LA, ALA and ARA may play a role (4, 7, 20, 42, 43, 44, 45, 46).

**Fig. 10:**
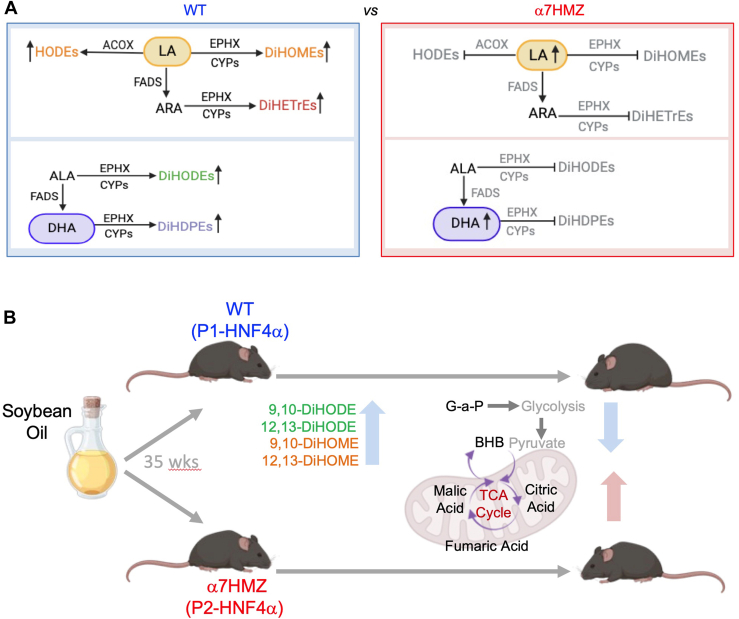
Proposed model for the role of HNF4α isoforms in diet-induced obesity. A: Comparison of LA and ALA metabolism in WT and α7HMZ mice fed the SO+CO diet. Gray text indicates lower levels of lipids and enzymes in α7HMZ livers. B: Potential drivers of SO- induced obesity in WT mice: increase in select oxylipins and decrease in metabolites involved in glycolysis and the TCA cycle. See text for details.

In this study, using global, untargeted metabolomics and an HNF4α exon swap (α7HMZ) mouse model that happens to express low levels of the enzymes (FADS1, EPHX1, ACOX1/2, CYP2Cs) that metabolize LA (and ALA) into oxylipins, we show that lower levels of LA/ALA diols in the liver correlate with resistance to soybean oil-induced obesity, reinforcing our previous finding that elevated levels of hepatic oxylipins in WT livers correlate positively with obesity (7) (Fig. 10A). Interestingly, the HNF4α exon swap mice, which are resistant to the SO-induced obesity, had high levels of LA in the liver indicating that LA itself does not correlate with obesity and supporting the notion that it is the ratio of LA/ALA to LA/ALA diols that drives SO-induced obesity. Furthermore, the epoxide precursors of the diols did not correlate with obesity in either the exon swap mice or when WT mice fed the soybean oil diet were treated with an epoxide inhibitor (sEHI) which increased epoxide levels but did not alter body weight. In contrast, the inhibitor had differential effects on the diols which allows us to further narrow the oxylipins that could be potential drivers of SO-induced obesity to four compounds—9,10-DiHODE, 12,13-DiHODE, 9,10-DiHOME, 12,13-DiHOME (Fig. 10B). It is noteworthy that these compounds include both omega-3 (DiHODEs) and omega-6 (DiHOMEs) oxylipins, despite the fact that omega-3 fatty acids are generally considered to be healthier than omega-6.

While oxylipins may be necessary to promote obesity, they are apparently not sufficient as the exon swap mice fed the low-fat diet had elevated levels of all four diols in their livers. Furthermore, we observed additional metabolic changes that could also contribute to obesity in the WT animals and the lean phenotype in the exon swap mice. For example, increased levels of glycerol-3-phosphate and β-hydroxybutyric acid (a major ketone body) in the α7HMZ mice fed the soybean diet suggests enhanced mitochondrial activity in the presence of P2-HNF4α. Furthermore, the WT mice, but not the exon swap mice, had reduced levels of key intermediates in the TCA cycle (citric acid, malic acid, and fumaric acid) on the soybean oil diet, suggesting reduced mitochondrial function and hence energy expenditure which could contribute to SO-induced obesity (Fig. 10B). Mitochondrial dysfunction is known to be an important feature of fatty liver disease, which often accompanies obesity (47, 48). A potential alteration in mitochondrial function could also be related to the oxylipins as the oxidized forms of LA in the mitochondria have been shown to impair the function of this key organelle (49).

The only other group of metabolites that were identified as a high impact factor in the metabolomics pathway analysis were aromatic amino acids (Phe, Trp, and Tyr) which were reduced in SO-fed WT but not in α7HMZ livers. This decrease, however, may not be driving the SO-induced obesity observed in our experiments as obese humans and mice tend to have higher levels of these amino acids, at least in the serum (50, 51). They could, however, be related to the fatty liver phenotype observed in the WT livers as aromatic amino acids (Phe, The, and Tyr) have been shown to ameliorate hepatic steatosis (52). Reduced fatty liver in the SO-fed exon swap mice compared to WT mice can also be explained by the lower levels of di- and tri-acylglycerols.

### Role for HNF4α in LA metabolism

The results presented here show that HNF4α isoforms are involved in LA/ALA metabolism via the CYP2C proteins. Our previously published RNAseq data from mice on the chow diet show that the low protein levels of the CYP2C enzymes in the α7HMZ livers is likely due to decreased expression of the whole *Cyp2c* locus. Additionally, ChIPseq data show HNF4α bound to the promoter region of all four genes locus in the livers of WT mice fed the chow diet (Supplemental Fig. 13) (16) suggesting that HNF4α directly regulates the expression of these genes. These findings are relevant in that LA is the endogenous ligand for HNF4α although the role of LA in HNF4α function is not clear—it does not significantly alter its ability to regulate transcription; rather, it seems to have a negative effect on its protein stability (8). Even more perplexing is why the alternate form of HNF4α in the liver would play a different role in LA metabolism than the predominant P1-HNF4α isoform in WT mice: P2-HNF4α is not anticipated to bind LA in a differential fashion to P1-HNF4α since they have identical ligand-binding domains. Furthermore, while the P2-HNF4α isoform is present during the neonatal period and decreases after birth (21), its expression is reactivated in adulthood under conditions of a high fat diet, fasting, alcoholic fatty liver and liver cancer (10). This suggests that these conditions may have different requirements for LA.

### Limitations and future directions

One limitation of this study is that we examined a single tissue (liver) and focused primarily on oxylipins for the follow up analysis. In addition to the liver, the HNF4α exon swap mice also have altered expression of the HNF4α isoforms in the kidney and the intestines where P1—but not P2-HNF4α is normally expressed (10). For example, we previously examined the impact of the soybean oil diet on colitis and found that not only did SO increase oxylipin levels in the gut but it also decreased the levels of beneficial endocannabinoids in both the host epithelial cells as well as the gut microbiota (4). We also previously examined the levels of oxylipins in the plasma of WT mice fed the soybean oil diet and found that, in contrast to the liver, they were not elevated compared to the low-fat control diet (7). This could suggest that tissues other than the liver may have taken up the oxylipins, such as adipose tissue (53).

Another limitation is that only male mice were used in this study, as female mice do not get obese on the soybean oil diet (our unpublished data, PD, JRE, KC, DSH, FMS). Nonetheless, others have shown that many oxylipins are impacted by sex and that male rats tend to have higher levels of oxylipins than female rats (54). Finally, our diet model is mouse, not human, although there are an increasing number of reports of diets consisting of ultra-processed foods (UPFs) being associated with obesity, and other diseases, in humans—seed oils with high omega-6, such as soybean oil, are often used in UPF (55, 56, 57, 58).

Future directions include determining by what mechanisms the oxylipins identified in this study are driving the obese phenotype—e.g., exactly what genes, protein function, organelles and/or cell systems are affected by the oxylipins. Analysis of other metabolites altered by the soybean and coconut oil diets, including those involved in mitochondrial function, also needs to be done. While our platform detected >3600 compounds, the vast majority are not annotated and hence were not considered in this study. Finally, α7HMZ livers had elevated levels of the anti-inflammatory fatty acid DHA as well as reduced levels of several pro-inflammatory cytokines in all three diets (Viv, CO, and SO), although the cytokines were not increased by the soybean oil diet in WT livers. Thus, interactions between oxylipins and other parameters associated with obesity, such as inflammation and mitochondrial function, need to be analyzed.

## Data availability

See Supplemental Tables 2 and 3 for all the datasets, including separate datasheets for primary metabolites, complex lipids, oxylipins and proteomics, cytokine results as well as information sheets for each platform. The raw metabolomics data has been deposited on Metabolomics Workbench (www.metabolomicsworkbench.org) under Project # PR000461. Proteomics data for liver tissue has been deposited in the proteomics repository Massive http://massive.ucsd.edu with an ID# MSV000081149 and can also be accessed via Proteome Exchange with a Proteome Exchange # PXD006681.

## Supplemental data

This article contains supplemental data (7, 20).

## Conflict of interest

The authors declare that they do not have any conflicts of interest with the content of this article.
