# Supplementary material for: P2-HNF4α alters linoleic acid metabolism and mitigates soybean oil-induced obesity: role for oxylipins
Source: J Lipid Res. 2025 Oct 28;66(12):100932. doi: 10.1016/j.jlr.2025.100932 (PMC12743519; doi:10.1016/j.jlr.2025.100932)
Supplement: Supplemental Materials [file mmc1.pdf]

Supplemental Materials For

P2-HNF4α Alters Linoleic Acid Metabolism and Mitigates Soybean Oil-Induced Obesity: Role for Oxylipins

Deol et al 2025

This file contains:

Supplemental Table 1
Supplemental Figures S1 to S13
Additional Supplemental Information: Details on the metabolomics pathway analysis (for Supplemental Figure S4A)

Supplemental Table 2 (metabolomic data and correlation plots) and Supplemental Table 3 (cytokine data, oxylipin data from sEHI experiment) are in separate Excel files.

Supplemental Table 1. Composition of diets used in this study.

A Fatty acid composition (%) of oils

	**Coconut**	**Soybean**
Lauric (12:0)	45	<0.05
Myristic (14:0)	17.5	0.07
Palmitic (16:0)	8.67	10.6
Stearic (18:0)	10.2	3.98
Oleic (18:1)	0.25	20.9
Linoleic (18:2 ω6)	<0.06	52.9
α-linolenic (18:3 ω3)	<0.06	6.54
ω6:ω3 (18:2/18:3)	n.a.	8.1

n.a., not applicable

B Composition of diets

	**Viv**	**CO**	**SO+CO**
**Nutrient**	**gm%**	**gm%**	**gm%**
Protein	23.9	20.1	20.1
Carbohydrate	48.7	53.4	53.4
Fat	5.0	21.5	21.5
kcal/gm	*3.36*	*4.87*	*4.87*
**Fat (kcal%)**	**13**	**40**	**40**
**Ingredient**		**gm**	**gm**
Casein, 80 Mesh		228	228
DL-Methionine		2	2
Maltodextrin 10		120	120
Corn Starch		480	480
Soybean Oil		25	115
Plenish Oil		0	0
Coconut Oil, Hydrogenated		220	130
Olive Oil		0	0
Lard		0	0
Mineral Mix S10001		40	40
Sodium Bicarbonate		10.5	10.5
Potassium Citrate, 1 H2O		4	4
Vitamin Mix V10001		10	10
Choline Bitartrate		2	2
**Total kcal%**		**5557**	**5557**

For complete chemical composition of the Viv chow see Laboratory Rodent Diet #5001 by LabDiet.

Supplemental Figure 1

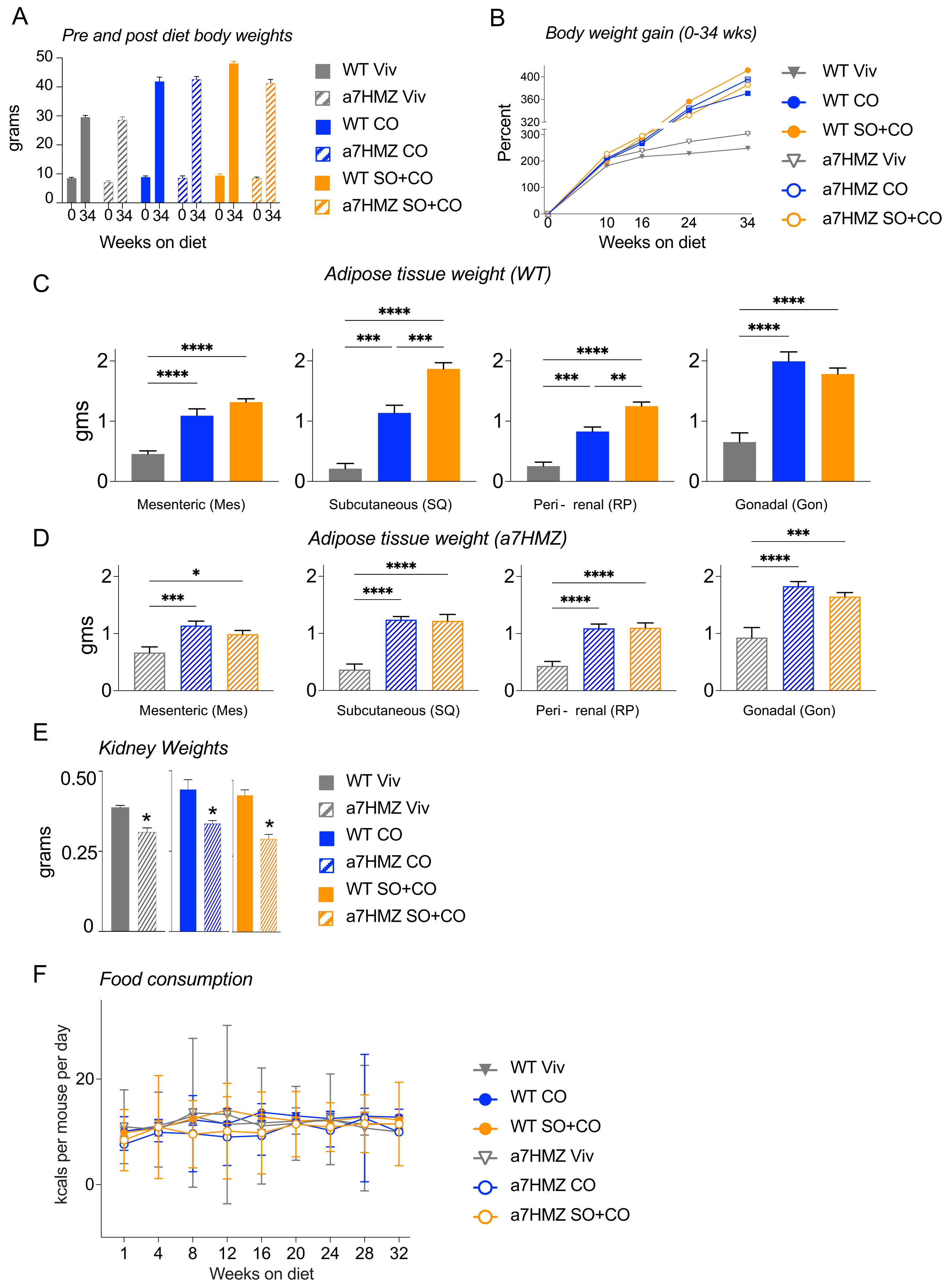

Supplemental Figure 1. Body weight, tissue weight and food consumption of male mice fed soybean oil and control diets.
A. Body weight comparison of WT (C57BL/6N) and HNF4α exon swap (α7HMZ) male mice at the start (at weaning, 0 weeks) and completion (34 weeks) of the indicated diets – low fat diet (Viv) and 40 kcal% fat diets: CO, coconut oil; SO + CO, soybean oil-enriched. **B.** Percent increase in body weight at 10, 16, 24 and 34 weeks on the diets. **C, D.** Average weight of white adipose tissues in WT (**C**) and a7HMZ (**D**) mice at harvest (35 weeks on diet). **E.** Average kidney weight at harvest. **F.** Average kcals consumed per mouse per day during the duration of the study on the indicated diets. Data are presented as ± SEM. Significantly different (*P<0.05, **P<0.01, ***P<0.001) by one-way ANOVA (Benjamini Hochberg post-hoc analysis). N = 9-13 mice per group.

Supplemental Figure 2

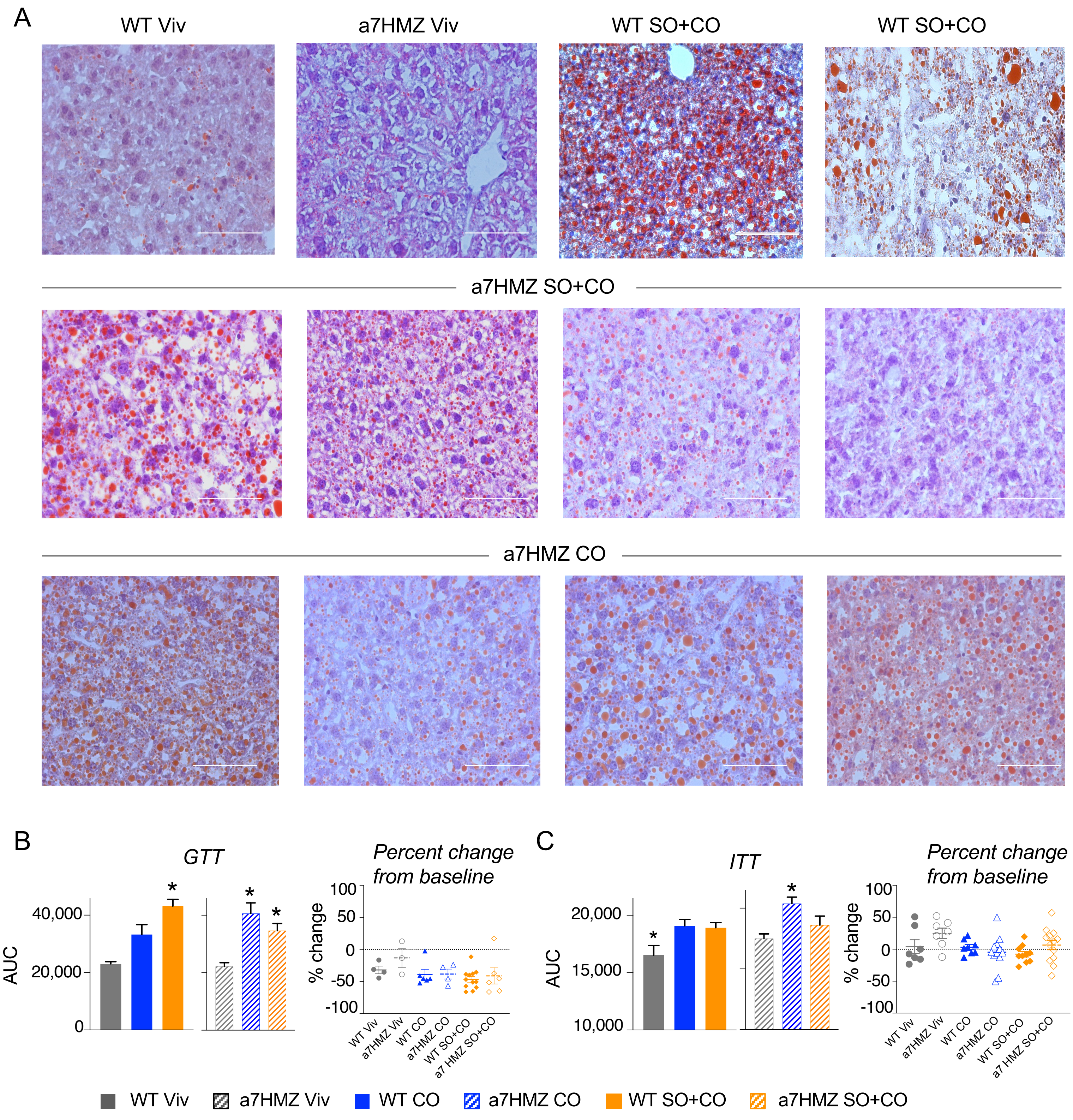

Supplemental Figure 2. Oil Red O staining of liver sections, GTT and ITT of male mice fed soybean oil and control diets A. Oil Red O staining of liver sections from WT and α7HMZ mice fed the various diets (35 weeks) as in Supplemental Figure 1. Scale bar is 100 microns. Images for WT CO not available, see References 7 and 20 for Oil Red O sections from similarly treated mice. **B.** Area under the curve (AUC) and percent change in glycemia at 120 min from baseline (0 min) for glucose tolerance test (GTT) of mice on diets for 20 weeks. **C.** Area under the curve (AUC) and percent change in glycemia at 90 min from baseline (0 min) for insulin tolerance test (ITT) of mice on diets for 33 weeks. **B-C**. N = 6–12 mice per group. Data are presented as ± SEM. *Significantly different (P<0.05) by one-way ANOVA (Benjamini Hochberg post-hoc analysis).

Supplemental Figure 3

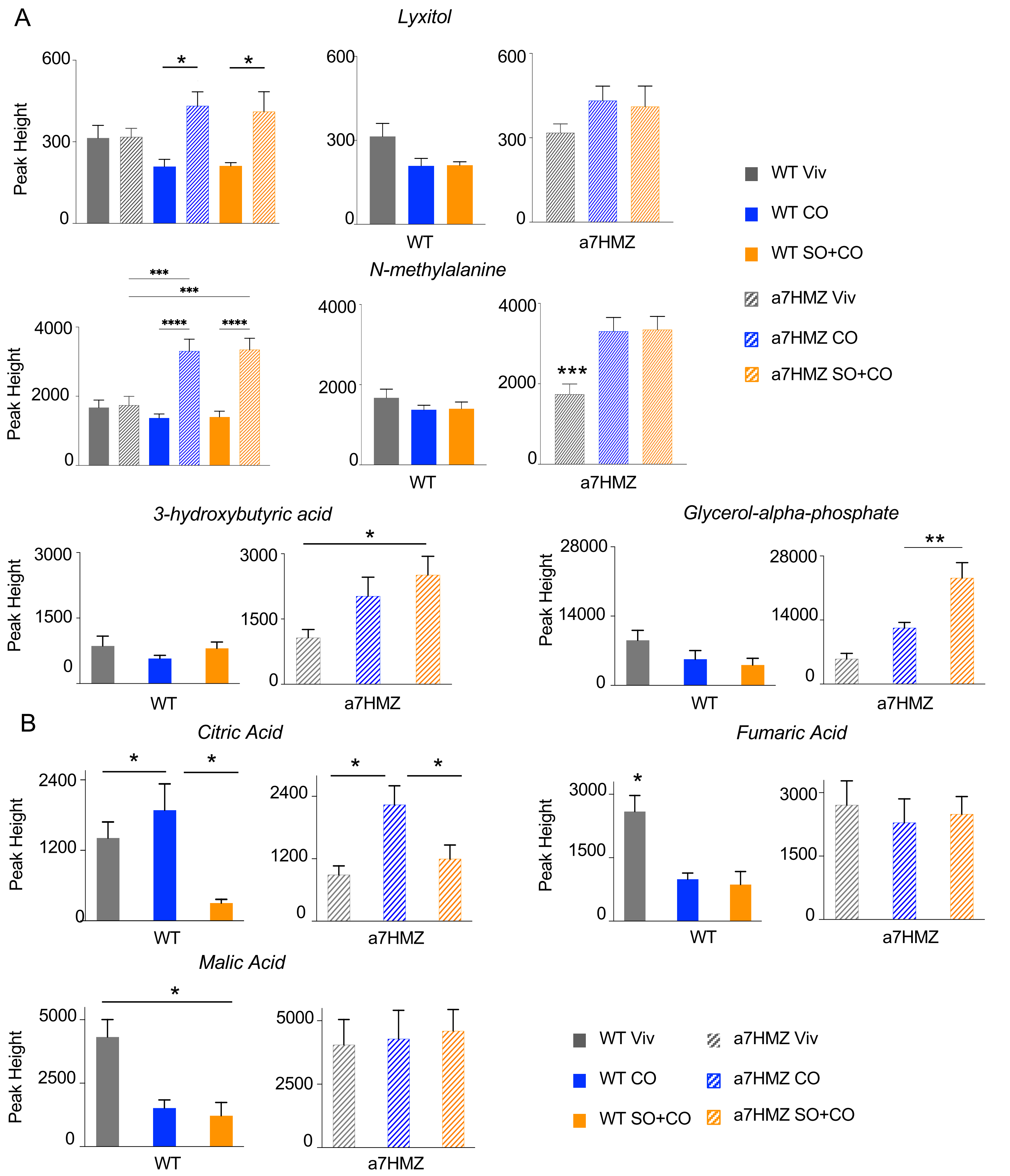

Supplemental Figure 3. Primary metabolite levels in livers of male mice fed soybean oil and control diets.
A, B. Levels of the indicated primary metabolites in the livers of mice fed the indicated diets for 35 weeks. See Figure 3 for graphs with WT and a7HMZ data presented together for 3-hydroxybutyric acid, glycerol-alpha-phosphate, citric acid, fumaric acid and malic acid. Data are presented as ± SEM. Significantly different (*P<0.05, **P<0.01, ***P<0.001, ****P<0.0001) by one-way ANOVA (Tukey post-hoc analysis). N = 7–8 mice per group.

Supplemental Figure 4

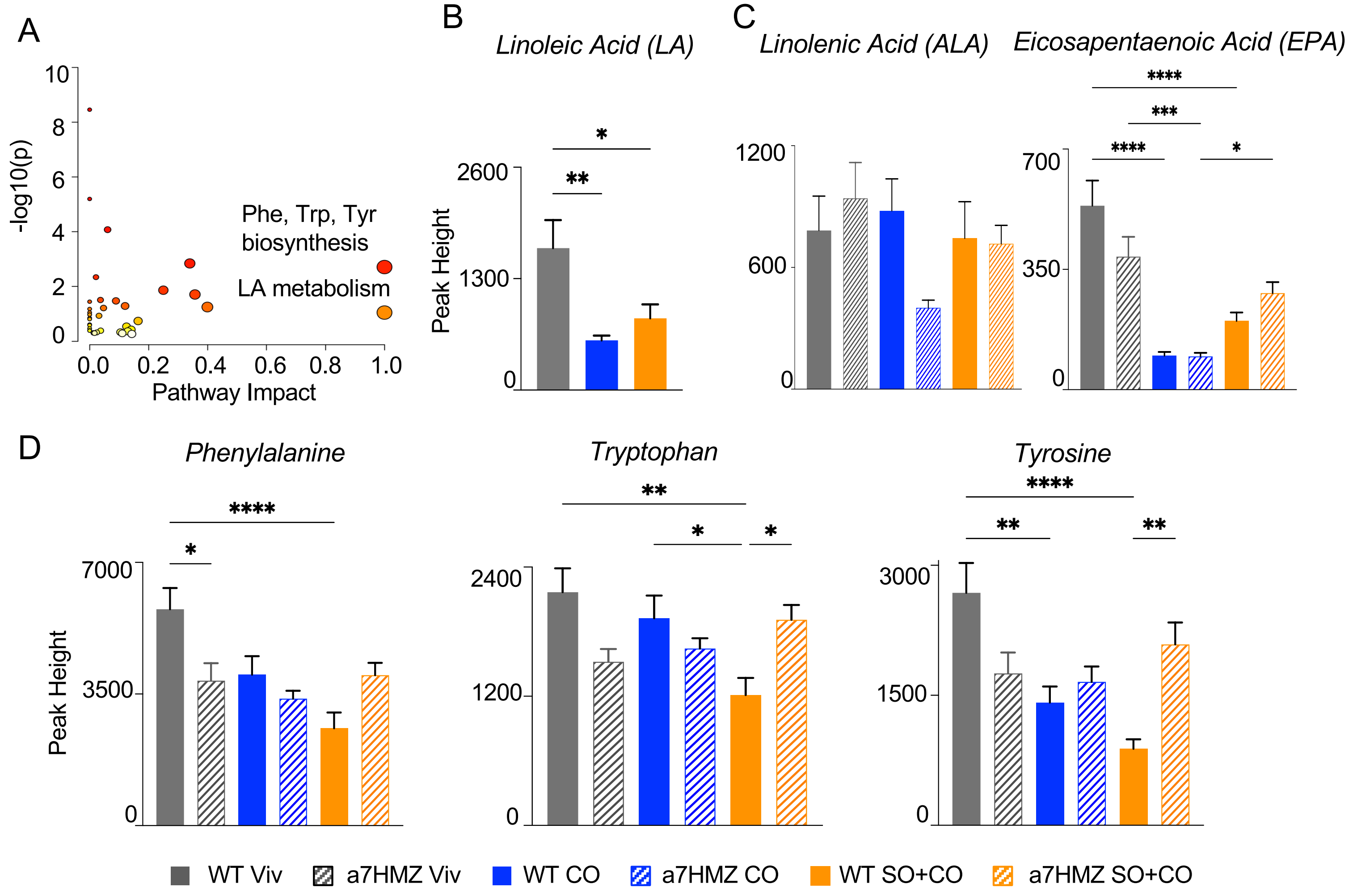

Supplemental Figure 4. Pathway analysis of primary metabolites and correlation analysis between body weight.

A. Pathway impact: pathway analysis (MetaboAnalyst 5.0 www.metaboanalyst.ca). Higher impact values represent the relative importance of the pathway. See supplementary document on Detailed Methodology for Pathway Analysis. **B,C,D.** Levels of the indicated primary metabolites in the livers of mice fed the respective diets for 35 weeks. Data are presented as ± SEM. Significantly different (*P<0.05, **P<0.01, ***P<0.001, ****P<0.0001) by one-way ANOVA (Tukey's post-hoc analysis). N = 7–8 mice per group.

Supplemental Figure 5

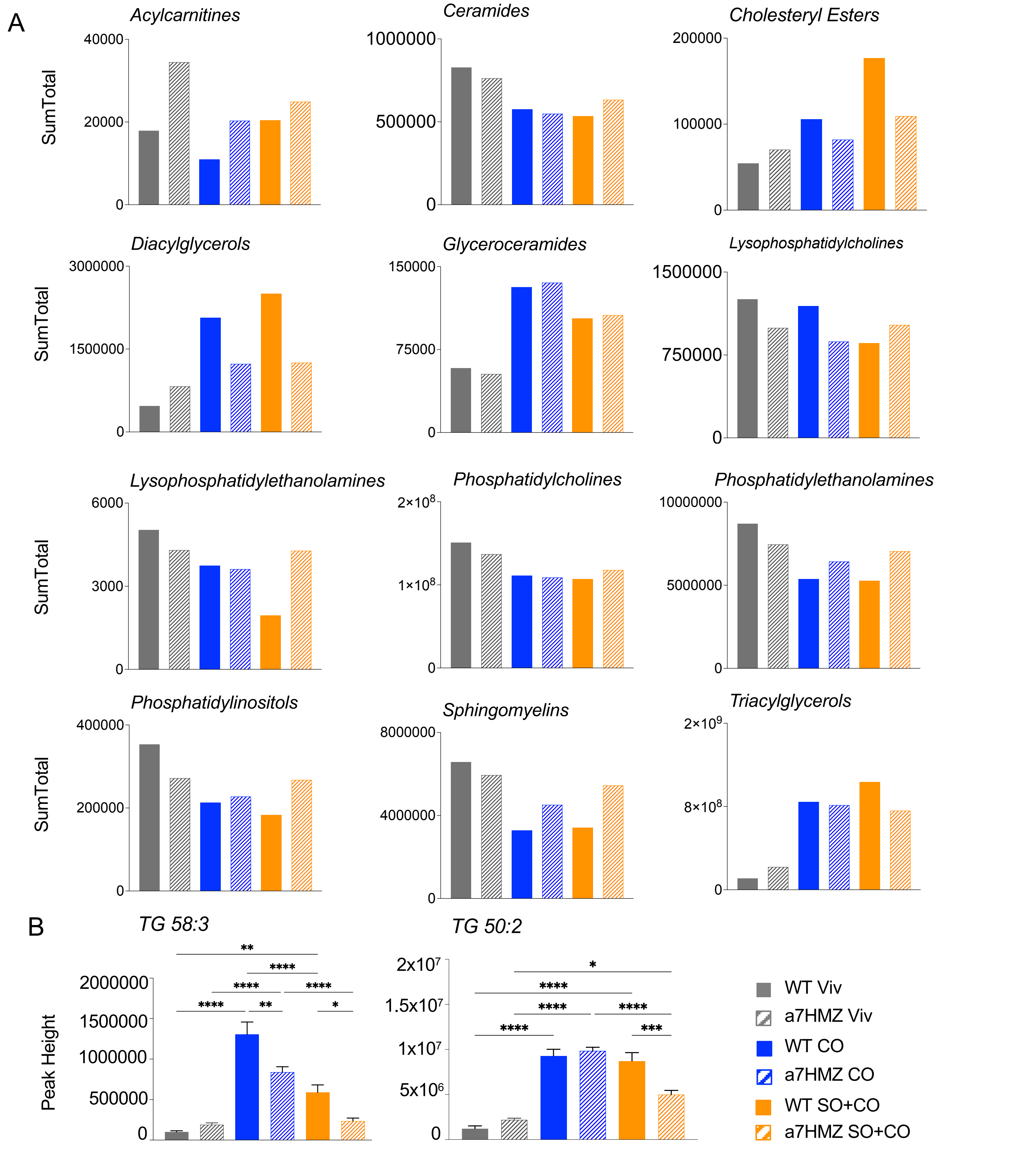

Supplemental Figure 5. Complex lipid levels in livers of male mice fed soybean oil and control diets. **A.** Cumulative levels of various complex lipid species belonging to the indicated lipid family in the livers of mice fed the indicated diets for 35 weeks. Bars represent the summed levels of individual lipid species within each lipid family, without replicate variance. Each species value reflects the average across 7-8 biological replicates; however, the totals are derived from a single LC–MS dataset. Error bars are not shown because the summed values are descriptive, not statistical. **B.** Levels of individual triacylglycerols in the livers of mice fed the indicated diets for 35 weeks. Data are presented as ± SEM. Significantly different (*P<0.05, **P<0.01, ***P<0.001, ****P<0.0001) by one-way ANOVA (Tukey post-hoc analysis). N = 7–8 mice per group.

Supplemental Figure 6

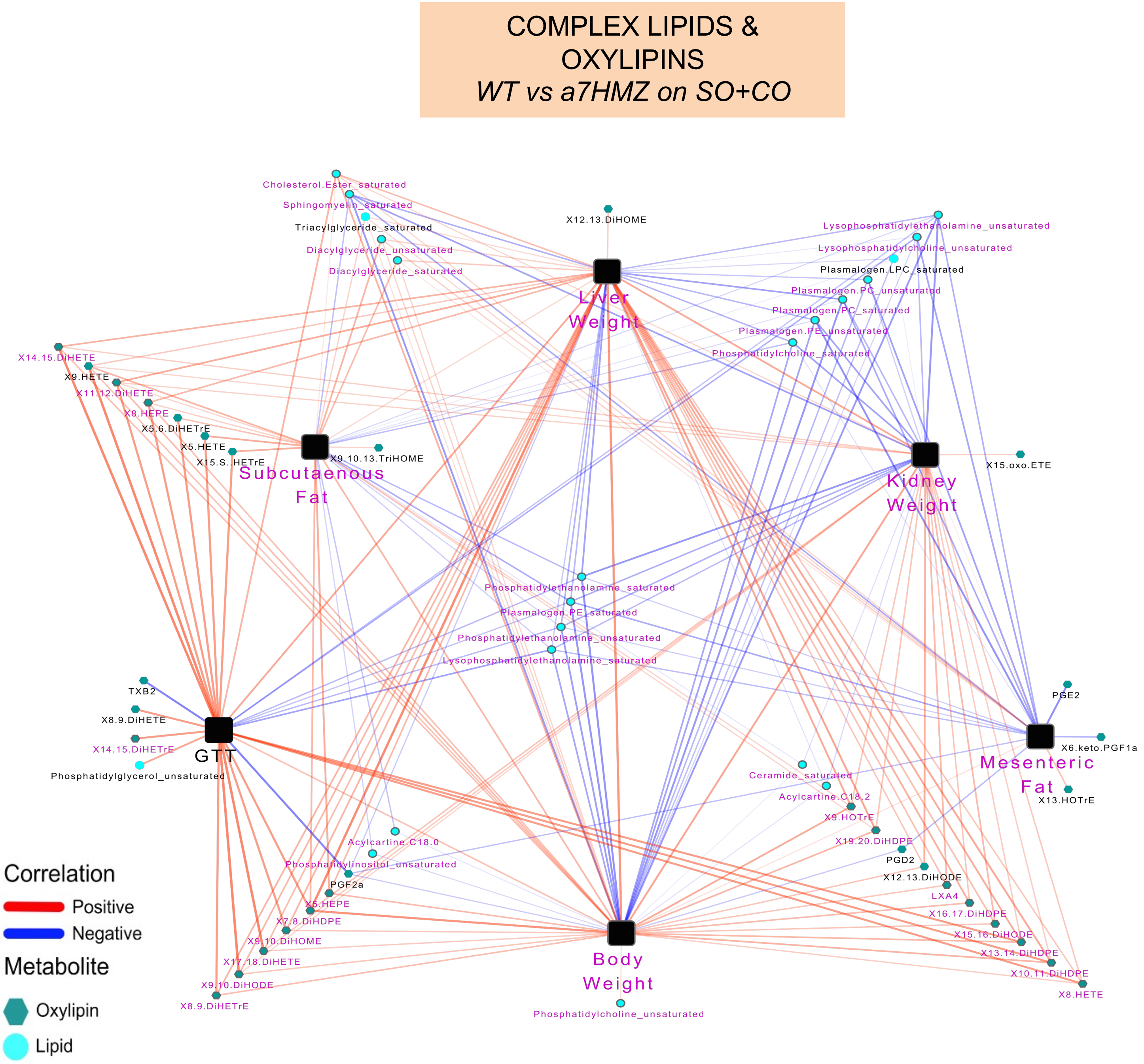

Supplemental Figure 6. Correlations between complex lipids, oxylipins and phenotypic measurements in male mice fed the soybean oil-enriched diet.
Correlations between phenotype and metabolites in livers of individual C57BL/6N (WT) and α7HMZ male mice fed the SO+CO diet. Edge colors depict correlation (red, positive; blue, negative) between metabolites and phenotypic measurements (black squares). Edge width represents the strength of the correlation. Node color and shape represent the different classes of metabolites. Only those samples with full meta-data and significant (P<0.05) Spearman's correlation coefficient were included. Networks were generated using Cytoscape as done previously (Deol et al 2017).

Supplemental Figure 7

Positively correlated oxylipins in livers of WT and a7HMZ male mice on SO+CO diet

	Body Weight	Kidney Weight	GTT
9-HOTrE (ALA)	✓		
12,13 DiHODE (ALA)	✓	✓	
15, 16 DiHODE (ALA)	✓	✓	
8 HETE (ALA)	✓	✓	✓
9,10 DiHODE (ALA)	✓	✓	✓
16,17 DiHDPE (DHA)	✓	✓	
19,20 DiHDPE (DHA)	✓		✓
7,8 DiHDPE (DHA)	✓	✓	✓
10, 11 DiHDPE (DHA)	✓	✓	✓
13, 14 DiHDPE (DHA)	✓	✓	✓
8 HEPE (EPA)	✓		✓
11,12 DiHETE (EPA)	✓		✓
14,15 DiHETE (EPA)	✓		✓
5 HEPE (EPA)	✓	✓	✓
14,15 DiHETE (EPA)			✓
17,18 DiHETE (EPA)	✓	✓	✓
8,9 DiHETE (EPA)			✓
9,10 DiHOME (LA)	✓	✓	✓
8,9 DiHETrE (AA)	✓	✓	✓
14,15 DiHETrE (AA)			✓
9-HETE (AA)	✓		
15-oxo-ETE (AA)		✓	
LXA4 (AA)	✓	✓	✓

Supplemental Figure 7. Overview of liver oxylipins from Supplemental Figure 6 that correlate positively with various metabolic phenotypes and are significantly different between WT vs α7HMZ mice on SO+CO diet for 35 weeks (P<0.05).

Supplemental Figure 8

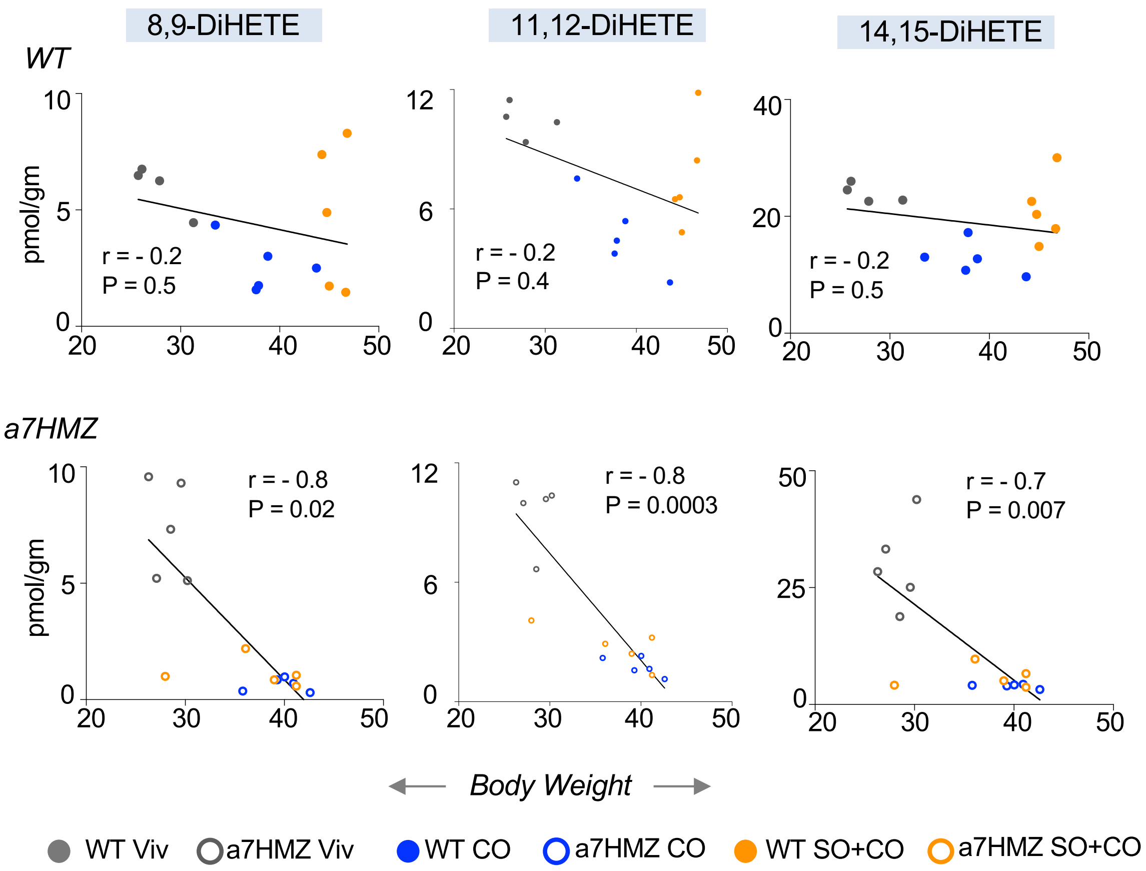

Supplemental Figure 8. Correlation analysis between body weight and DiHETEs in livers of male mice fed soybean oil and control diets.
Correlation between body weight and concentration of liver DiHETEs of individual mice. Spearman correlation coefficient (r) and P-value for each correlation is indicated on each graph. N = 5 mice per group.

Supplemental Figure 9

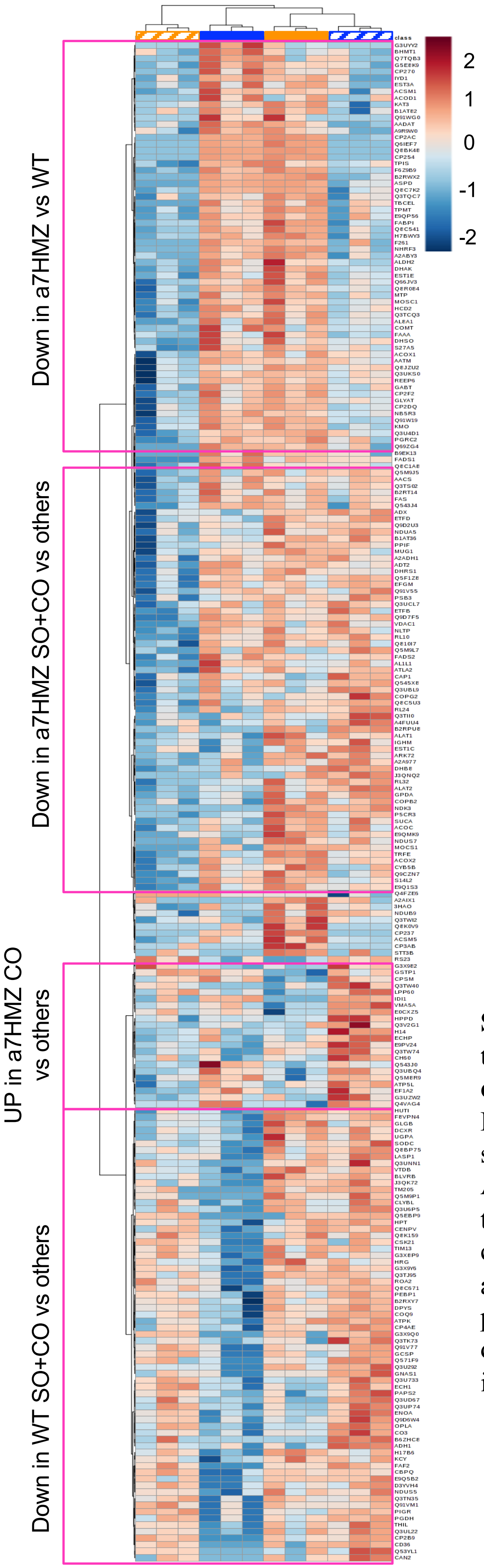

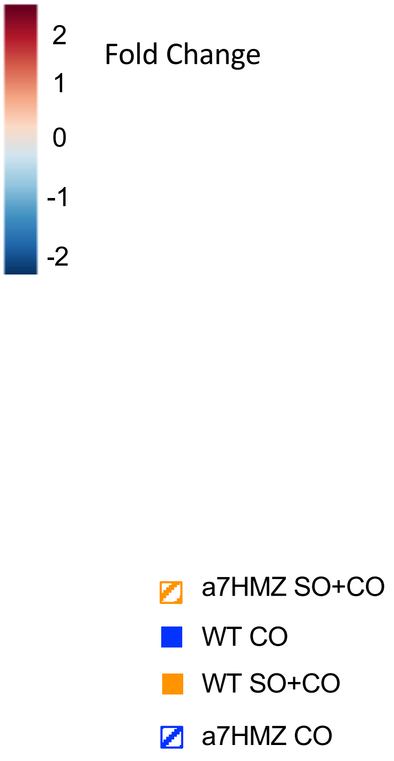

Supplemental Figure 9. Proteomic analysis of the liver reveals changes induced by both coconut and soybean oil enriched diets. Heatmap showing liver proteins that are significantly different ($P<0.05$ by one-way ANOVA) between diets. Values were log10 transformed; hierarchical clustering was calculated on Elucidean Distance with Wards agglomeration. Heatmap was generated with pheatmap package in R. Pink boxes drawn to demarcate groups of proteins up or down in indicated treatment. N = 3 livers per group.

Supplemental Figure 10

	WT SO+CO	a7 SO+CO	WT CO	a7 CO
Cyp 1a2	28.3	26	36.7	55.7
Cyp 27a1	26.7	16	33.7	26.7
**Cyp 2a12**	35.3	0	23.7	0.3
Cyp 2a4	0.7	0.3	0.3	0.3
**Cyp 2a5**	10.7	6.3	2	4.7
Cyp 2b9	4.7	8.3	2	9
**Cyp 2c37**	5	0	1.7	0
**Cyp 2c38**	35	2.3	30.7	4.7
**Cyp 2c44**	11	0	7.7	0
**Cyp 2c54**	35.3	0	26.3	0
Cyp 2c67	10.3	6.7	12	13.7
Cyp 2c70	3	0	11.3	1.3
Cyp 2d10	24.7	16.7	24	16
Cyp 2d22	13.3	8.7	11.7	10.7
**Cyp 2d26**	118	74.3	116.7	85
Cyp 2d9	13.7	12	15.3	13
Cyp 2e1	56.3	34.7	46.3	60.7
**Cyp 2f2**	46.7	20.3	45	28.7
Cyp 2j12	4.3	4	6.3	6.3
**Cyp 3a11**	1.7	0.3	0	0
Cyp 3a13	15.3	8	10.7	11.7
Cyp 3a25	2.3	3	0	2
**Cyp 3a41a**	15	6	1	2
Cyp 4a10	3.7	0.3	2.3	6
**Cyp 4a12a**	3.7	5.7	13.7	12.3
**Cyp 4a14**	9.7	7	2.3	19.3
Cyp 4f14	1.7	0.3	3	1.3
Cyp 4v3	9.3	6.3	6.3	8.3
Cyp 7b1	2.7	0	6.3	2
Cyp 8b1	4	0	1.7	0
Cyp 2c68	0	0.7	0.7	1

Color scale

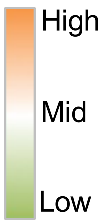

Supplemental Figure 10. Proteomic analysis reveals that livers of α7HMZ mice have greatly decreased levels of many CYP450 enzymes.

Heatmap (generated with Excel) showing relative levels of CYP450 enzymes in livers of WT and α7HMZ mice fed either the SO+CO or CO diets. Red font, absolute values of CYPs plotted in Figure 7 in the main text. N = 3 livers per group.

Supplemental Figure 11

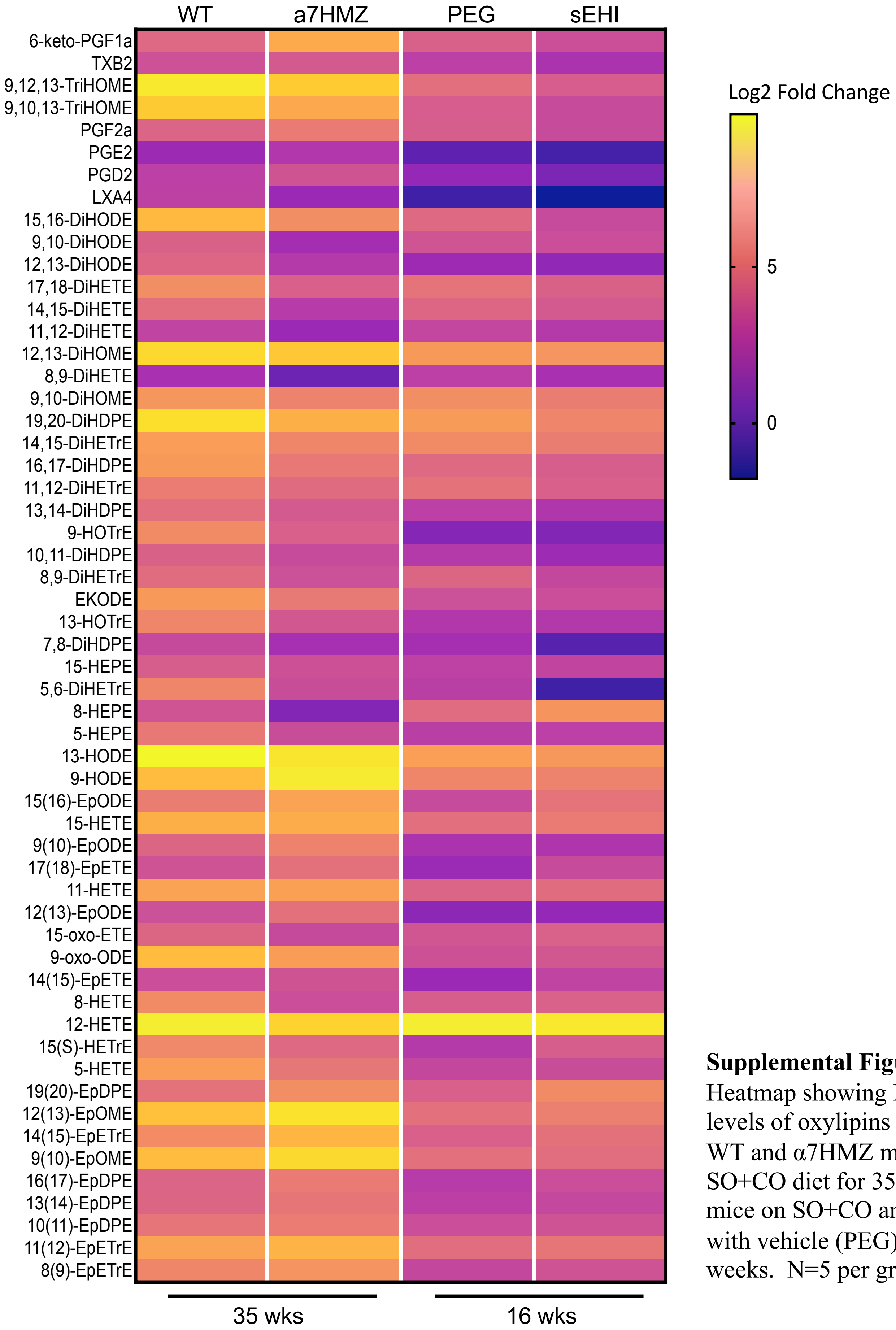

Supplemental Figure 11. Heatmap showing Log2 fold levels of oxylipins in livers of WT and α7HMZ mice fed the SO+CO diet for 35 weeks and WT mice on SO+CO and treated either with vehicle (PEG) or sEHI for 16 weeks. N=5 per group.

Supplemental Figure 12

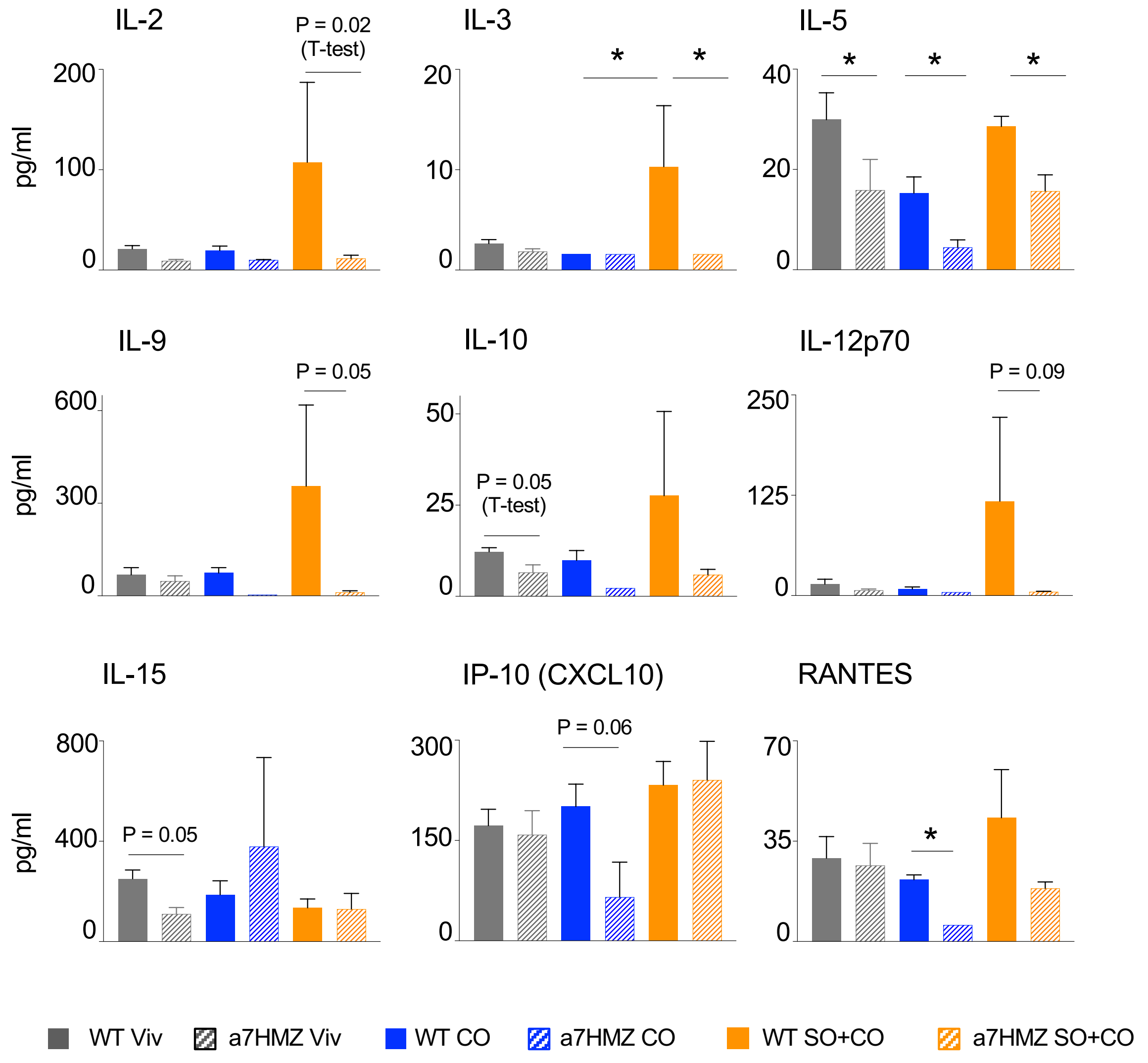

Supplemental Figure 12. Levels of cytokines as measured by multiplex cytokine analysis of livers of mice on indicated diets for 35 weeks. Significantly different (* P< 0.05). Significant differences and P-values are based on one-way ANOVA of all groups; P-values from separately conducted Student's T-test for IL-2 and IL-10 are indicated in the respective graphs. Data are presented as ± SEM. N = 4 mice per group. See Supplemental Table 3 for values of all cytokines analyzed.

Supplemental Figure 13

A RNAseq from liver of WT and a7HMZ males fed Viv chow

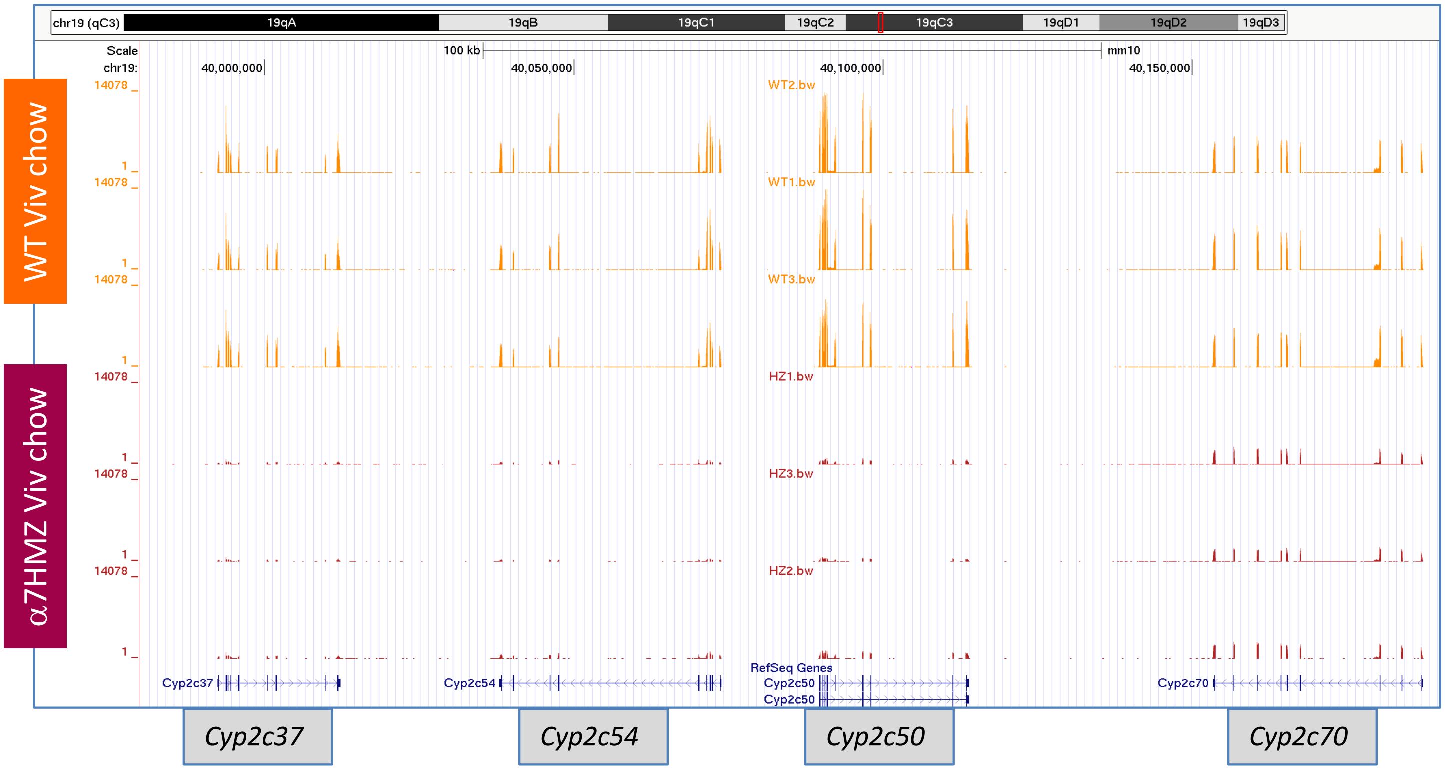

B HNF4α ChIPseq from liver of WT males fed Viv chow

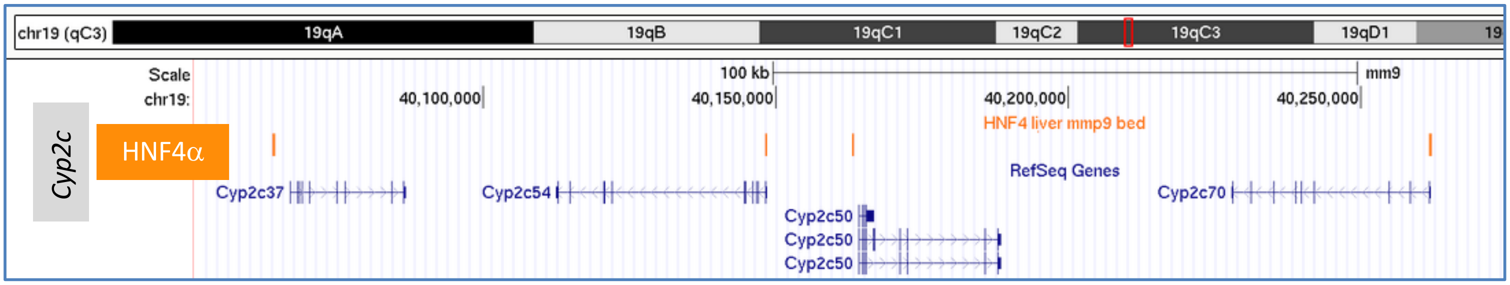

Supplemental Figure 13. HNF4α regulates the mouse *Cyp2c* locus.
A. UCSC Genome Browser view of RNAseq reads at the *Cyp2c* locus from the livers of WT and α7HMZ adult male mice on the low fat diet (Viv chow) showing a nearly complete lack of expression of *Cyp2c37*, *Cyp2c54* and *Cyp2c50* and greatly reduced levels of *Cyp2c70* in α7HMZ livers, consistent with the nondetectable levels of CYP2c37, CYP2c54 and CYP2c70 protein in α7HMZ livers on the SO+CO diet (see Supplemental Figure 10). **B.** UCSC Genome Browser view of ChIPseq reads at the *Cyp2c* locus showing that HNF4α binds the upstream regulatory region of all four *Cyp2c* genes in livers of WT adult male mice on the low-fat diet, suggesting direct regulation of HNF4α of this locus.

Additional Supplemental Information

Detailed methodology for Pathway Analysis depicted in Supplemental Figure 4A

Metabolomic Data Analysis with MetaboAnalyst 5.0

Name: guest3216487040574943840

May 16, 2023

1 Background

The Pathway Analysis module combines results from powerful pathway enrichment analysis with pathway topology analysis to help researchers identify the most relevant pathways involved in the conditions under study.

There are many commercial pathway analysis software tools such as Pathway Studio, MetaCore, or Ingenuity Pathway Analysis (IPA), etc. Compared to these commercial tools, the pathway analysis module was specifically developed for metabolomics studies. It uses high-quality KEGG metabolic pathways as the backend knowledgebase. This module integrates many well-established (i.e. univariate analysis, over-representation analysis) methods, as well as novel algorithms and concepts (i.e. Global Test, GlobalAncova, network topology analysis) into pathway analysis. Another feature is a Google-Map style interactive visualization system to deliver the analysis results in an intuitive manner.

2 Data Input

The Pathway Analysis module accepts either a list of compound labels (common names, HMDB IDs or KEGG IDs) with one compound per row, or a compound concentration table with samples in rows and compounds in columns. The second column must be phenotype labels (binary, multi-group, or continuous). The table is uploaded as comma separated values (.csv).

3 Compound Name Matching

The first step is to standardize the compound labels used in user uploaded data. This is a necessary step since these compounds will be subsequently compared with compounds contained in the pathway library. There are three outcomes from the step - exact match, approximate match (for common names only), and no match. Users should click the textbfView button from the approximate matched results to manually select the correct one. Compounds without match will be excluded from the subsequently pathway analysis.

Table 1 shows the conversion results. Note: *1* indicates exact match, *2* indicates approximate match, and *0* indicates no match. A text file contain the result can be found the downloaded file *name_ map.csv*

Table 1: Result from Compou

	Query	Match	HMDB	PubChem	KEGG	SMILES
1	2-hydroxybutanoic acid	2-Hydroxybutyric acid	HMDB0000008	11266	C05984	CCC(C(=O)O)O
2	3-aminoisobutyric acid	3-Aminoisobutanoic acid	HMDB0003911	64956	C05145	CC(CN)C(=O)O
3	3-hydroxybutyric acid	3-Hydroxybutyric acid	HMDB0000357	441	C01089	CC(CC(=O)O)O
4	adenine	Adenine	HMDB0000034	190	C00147	C1=NC2=C(N1)C(=NC=N2)N
5	alanine-alanine	NA	NA	NA	NA	NA
6	alpha-aminoadipic acid	Aminoadipic acid	HMDB0000510	469	C00956	C(CC(C(=O)O)N)CC(=O)O
7	aminomalonate	Aminomalonic acid	HMDB0001147	100714	C00872	C(C(=O)O)(C(=O)O)N
8	arachidic acid	Arachidic acid	HMDB0002212	10467	C06425	CCCCCCCCCCCCCCCCCCC
9	aspartic acid	L-Aspartic acid	HMDB0000191	5960	C00049	C([C@@H](C(=O)O)N)C(=O)

10	beta-alanine	Beta-Alanine	HMDB0000056	239	C00099	C(CN)C(=O)O
11	citric acid	Citric acid	HMDB0000094	311	C00158	C(C(=O)O)C(CC(=O)O)(C(=
12	digalacturonic acid	Digalacturonic acid	HMDB0039721	6857565	C02273	OC1OC(C(OC2OC(C(O)C(O)
13	doconexent NIST	NA	NA	NA	NA	NA
14	ethanolamine	Ethanolamine	HMDB0000149	700	C00189	C(CO)N
15	fumaric acid	Fumaric acid	HMDB0000134	444972	C00122	C(=C/C(=O)O)\C(=O)O
16	glutamine	L-Glutamine	HMDB0000641	5961	C00064	C(CC(=O)N)[C@@H](C(=O)O
17	glycerol-alpha-phosphate	NA	NA	NA	NA	NA
18	hexonic acid	Gluconic acid	HMDB0000625	10690	C00257	C([C@H]([C@H]([C@@H]([C@H
19	inosine	Inosine	HMDB0000195	6021	C00294	C1=NC(=O)C2=C(N1)N(C=N
20	isoleucine	L-Isoleucine	HMDB0000172	6306	C00407	CC[C@H](C)[C@@H](C(=O)O)
21	lactitol	NA	NA	NA	NA	NA
22	leucine	L-Leucine	HMDB0000687	6106	C00123	CC(C)C[C@@H](C(=O)O)N
23	linoleic acid	Linoleic acid	HMDB0000673	5280450	C01595	CCCCC/C=C\C/C=C\CCCC
24	lyxitol	NA	NA	NA	NA	NA
25	maleimide	NA	NA	NA	NA	NA
26	malic acid	Malic acid	HMDB0000744	525	C03668	C(C(C(=O)O)O)C(=O)O
27	maltose	Maltose	HMDB0037138	439341	C00897	OC[C@H]1O[C@H](O[C@H]2[C
28	mannose	D-Mannose	HMDB0000169	18950	C00936	C([C@@H]1[C@H]([C@@H]([C
29	methionine	L-Methionine	HMDB0000696	6137	C00073	CSCC[C@@H](C(=O)O)N
30	myo-inositol	myo-Inositol	HMDB0000211		C00137	O[C@H]1[C@H](O)[C@@H](O)[
31	N-methylalanine	N-Methylalanine	HMDB0094692	5288725	C02721	[H][C@@](C)(NC)C(O)=O
32	ornithine	Ornithine	HMDB0000214	6262	C00077	C(C[C@@H](C(=O)O)N)CN
33	phenylalanine	L-Phenylalanine	HMDB0000159	6140	C00079	C1=CC=C(C=C1)C[C@@H](C
34	phosphate	Phosphate	HMDB0001429	57424078	C00009	[O-]P(=O)([O-])[O-]
35	phosphogluconic acid	NA	NA	NA	NA	NA
36	pyrophosphate	Pyrophosphate	HMDB0000250	644102	C00013	[O-]P(=O)([O-])OP(=O)([O-])[
37	ribose	D-Ribose	HMDB0000283	5779	C00121	C([C@@H]1[C@H]([C@H](C(O1
38	serine	Serine	HMDB0062263	5951	C00716	N[C@@H](CO)C(O)=O
39	threonine	L-Threonine	HMDB0000167	6288	C00188	C[C@H]([C@@H](C(=O)O)N)C
40	tryptophan	L-Tryptophan	HMDB0000929	6305	C00078	C1=CC=C2C(=C1)C(=CN2)C
41	tyrosine	L-Tyrosine	HMDB0000158	6057	C00082	C1=CC(=CC=C1C[C@@H](C(
42	UDP-glucuronic acid	Uridine diphosphate glucuronic acid	HMDB0000935	17473	C00167	C1=CN(C(=O)NC1=O)[C@H]
43	uridine-5'-monophosphate	Uridine 5'-monophosphate	HMDB0000288	6030	C00105	C1=CN(C(=O)NC1=O)[C@H]
44	valine	L-Valine	HMDB0000883	6287	C00183	CC(C)[C@@H](C(=O)O)N
45	xanthine	Xanthine	HMDB0000292	1188	C00385	C1=NC2=C(N1)C(=O)NC(=O

4 Pathway Analysis

In this step, users are asked to select a pathway library, as well as specify the algorithms for pathway enrichment analysis and pathway topology analysis.

4.1 Pathway Library

There are 15 pathway libraries currently supported, with a total of 1173 pathways :

- Homo sapiens (human) [80]
- Mus musculus (mouse) [82]
- Rattus norvegicus (rat) [81]
- Bos taurus (cow) [81]
- Danio rerio (zebrafish) [81]
- Drosophila melanogaster (fruit fly) [79]
- Caenorhabditis elegans (nematode) [78]
- Saccharomyces cerevisiae (yeast) [65]
- Oryza sativa japonica (Japanese rice) [83]
- Arabidopsis thaliana (thale cress) [87]
- Escherichia coli K-12 MG1655 [87]
- Bacillus subtilis [80]
- Pseudomonas putida KT2440 [89]
- Staphylococcus aureus N315 (MRSA/VSSA)[73]
- Thermotoga maritima [57]

Your selected pathway library code is **mmu** (KEGG organisms abbreviation).

4.2 Over Representation Analysis

Over-representation analysis tests if a particular group of compounds is represented more than expected by chance within the user uploaded compound list. In the context of pathway analysis, we are testing if compounds involved in a particular pathway are enriched compared to random hits. MetPA offers two of the most commonly used methods for over-representation analysis:

- Fishers'Exact test
- Hypergeometric Test

Please note, MetPA uses one-tailed Fisher's exact test which will give essentially the same result as the result calculated by the hypergeometric test.

The selected over-representation analysis method is **Hypergeometric test.**

4.3 Pathway Topology Analysis

The structure of biological pathways represent our knowledge about the complex relationships among molecules within a cell or a living organism. However, most pathway analysis algorithms fail to take structural information into consideration when estimating which pathways are significantly changed under conditions of study. It is well-known that changes in more important positions of a network will trigger a more severe impact on the pathway than changes occurred in marginal or relatively isolated positions.

The pathway topology analysis uses two well-established node centrality measures to estimate node importance - **degree centrality** and **betweenness centrality**. Degree centrality is defined as the number of links occurred upon a node. For a directed graph there are two types of degree: in-degree for links come from other nodes, and out-degree for links initiated from the current node. Metabolic networks are directed graph. Here we only consider the out-degree for node importance measure. It is assumed that nodes upstream will have regulatory roles for the downstream nodes, not vice versa. The betweenness centrality measures the number of shortest paths going through the node. Since the metabolic network is directed, we use the relative betweenness centrality for a metabolite as the importance measure. The degree centrality measure focuses more on local connectivities, while the betweenness centrality measure focuses more on global network topology. For more detailed discussions on various graph-based methods for analyzing biological networks, please refer to the article by Tero Aittokallio, T. et al. [1]

Please note, for comparison among different pathways, the node importance values calculated from centrality measures are further normalized by the sum of the importance of the pathway. Therefore, the total/maximum importance of each pathway is 1; the importance measure of each metabolite node is actually the percentage w.r.t the total pathway importance, and the pathway impact value is the cumulative percentage from the matched metabolite nodes.

Your selected node importance measure for topological analysis is **relative betweenness centrality**.

5 Pathway Analysis Result

The results from pathway analysis are presented graphically as well as in a detailed table.

A Google-map style interactive visualization system was implemented to facilitate data exploration. The graphical output contains three levels of view: **metabolome view**, **pathway view**, and **compound view**. Only the metabolome view is shown below. Pathway views and compound views are generated dynamically based on your interactions with the visualization system. They are available in your downloaded files.

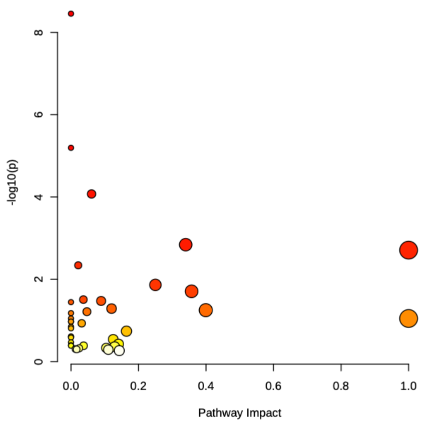

Figure 1: Summary of Pathway Analysis

[1]Tero Aittokallio and Benno Schwikowski. *Graph-based methods for analyzing networks in cell biology*, Briefings in Bioinformatics 2006 7(3):243-255
